# Field study of a web service for stimulating the positive side of stress: entrepreneurs’ experiences and design implications

**DOI:** 10.1186/s12911-019-0909-6

**Published:** 2019-10-28

**Authors:** Päivi Heikkilä, Elina Mattila, Mari Ainasoja

**Affiliations:** 1 VTT Technical Research Centre of Finland LTD, P. O. Box 1300, FI-33101 Tampere, Finland; 2 Tampere University, Tampere, Finland

**Keywords:** Human-centred design, Positive stress, Eustress, Well-being, Entrepreneurs, User experience

## Abstract

**Background:**

Digital services have been found promising in managing different aspects of health, also stress. We developed a web service for cultivating the positive side of stress based on the stress experiences of entrepreneurs. In this paper, we present a field study conducted to evaluate the user acceptance and the user experience of the developed service.

**Methods:**

Twenty-two participants, working as entrepreneurs or having an entrepreneurial-type job description, used the web service for 6 weeks. User experiences were collected from all participants with electronic questionnaires, and 10 participants were interviewed to gain deeper understanding and to formulate design implications. In addition, usage logs of the web service were analysed to assess how actively the participants used the service and a pre and post questionnaires on stress and work engagement were conducted to evaluate the preliminary effectiveness of the service.

**Results:**

The usage activity of the service was relatively low, on average, the service was used on 3 days and a total of 101 min. During the usage period, the participants’ negative stress measured by the perceived stress scale decreased and their self-reported positive stress experiences had increased. The participants considered the positive perspective to stress useful. In the Eustress Toolbox service, the users appreciated especially the off-line and reflection exercises, as well as the quotations from peers, but the design should have supported more active triggering to use the service.

**Conclusions:**

Based on user experience, we propose four design implications: *Integrate the service into the daily hassle of entrepreneurs, Provide personal guidance while maintaining a possibility to explore, Recognise the user’s progress and accomplishments in a meaningful way* and *Support implicit learning from peer entrepreneurs*.

**Trial registration:**

ISRCTN14739582, Sept 3 2019, retrospectively registered.

## Background

Stress is a significant challenge in the working life. It causes serious health problems for individuals ([13]; Kivimäki et al. 2006) and has been associated with societal costs due to, for example, absenteeism and loss of productivity [11]. Entrepreneurial work may subject individuals to higher levels of stress due to the risks in business activities [38], a higher-than-average need for achievement [26], heavy workloads and a self-established role in the organization [18]. In Finland, employers are required by law to organize preventive occupational health services for their employees. However, for entrepreneurs and the self-employed, the arrangement of occupational health services is voluntary, often leading to a lack of preventive care [48].

On the other hand, many entrepreneurs have traits and skills that help them in the face of stress, such as high stress tolerance [12], a high internal locus of control [39], or high levels of psychological capital (a construct of self-efficacy, optimism, hope and resilience) [3]. According to a study by Cardon and Patel [5], self-employed people do experience greater stress than employees but as it has a positive effect on their income, they may have a less negative attitude to stress. Several studies confirm [3, 5, 46] that coping with and managing stress effectively are critical skills to entrepreneurs and for survival and success of their companies. These findings on successful entrepreneurs’ strengths in managing stress indicate that other entrepreneurs and employees could learn from their ways of managing stress. The growing research interest in entrepreneurial mindset [20, 23, 33] and self-leadership [31, 35] imply that work in general has become increasingly entrepreneurial in nature.

The positive side of stress has been acknowledged already for several decades [27, 40], but this perspective is typically excluded from stress research and design experiments related to managing stress. The term eustress refers to the beneficial side of stress [40], or more exactly, to the positive response to stressors [44]. The term was introduced by Selye [40], who divided the stress concept into the positive and harmful sides of it: eustress and distress. This has paved the way to the current holistic understanding of stress, which emphasizes that the stressor type itself does not lead to eustress or distress, but a person may interpret a stressor as a positive challenge or a negative threat [28, 44] and that eustress and distress are not mutually exclusive [44]. The savouring of eustress has been suggested to have the potential to increase the well-being of an individual [17, 44]. The concept of work engagement is closely related to eustress, but where work engagement is seen as a relatively stable state, eustress is a short-term response to stressors [28, 44]. Work engagement may be conceptualized as one of the positive outcomes of experiencing eustress (Hargrove, Becker, and Hargrove, 2015).

Digital interventions have increasingly been used for disseminating interventions for preventing stress-related problems and educating individuals on skills related to stress management. Several studies have reported promising stress-related outcomes with web-based programs and mobile applications (e.g. [2, 19, 30, 47, 49]). There are also examples of programs focusing on building positive resources such as personal strengths, positive emotions, self-efficacy, and happiness [29, 34, 37]. However, to the authors’ knowledge, interventions focusing specifically on building skills to foster positive stress have not been reported. Furthermore, most studies have been conducted with organizationally employed people from workers to managers, and they may exclude some aspects relevant in the entrepreneurial context.

We have previously reported the development and implementation of a web-based program for practicing eustress-related skills, the Eustress Toolbox [22]. The program was developed by interviewing a group of entrepreneurs and analysing their personal experiences and techniques to foster eustress. In this paper, we present a field study conducted to evaluate the user experience of the Eustress Toolbox among a group of people working as entrepreneurs or having an entrepreneur-type work description. Our research objectives are to study the acceptance and usage of the Eustress Toolbox web service, and based on the findings, provide design implications to support the design of digital well-being services especially in the entrepreneurial context.

The specific research questions were:

1. How is the Eustress Toolbox accepted and experienced by entrepreneurs and people doing entrepreneur-like work?
2. Does the use of the service affect the users’ experiences of stress and work engagement and are there perceived benefits from using the service?
3. How should the service be developed and what design implications can be derived?

## Methods

### Eustress toolbox

The Eustress Toolbox is a prototype web service, which enables users to familiarize themselves with the phenomenon of positive stress and introduces different methods to stimulate eustress in one’s daily life. The service is primarily targeted at entrepreneurs and people having an entrepreneur-like job. The aim of the Eustress Toolbox is to give inspiration and exercises to change one’s ways of thinking and working in a way that would contribute to experiencing positive stress. The toolbox is designed for preventive, temporary and selective use: users can choose the parts that are useful for them and do the exercises from the selected parts. By providing the users knowledge of the phenomenon of eustress, quotes illustrating other entrepreneurs’ experiences, reflection exercises and off-line exercises, they are guided and encouraged to increase positive stress in their daily work and life. The toolbox can be used with mobile devices, but it is not optimized for mobile use. The content is mainly in textual format.

**Fig. 1:**
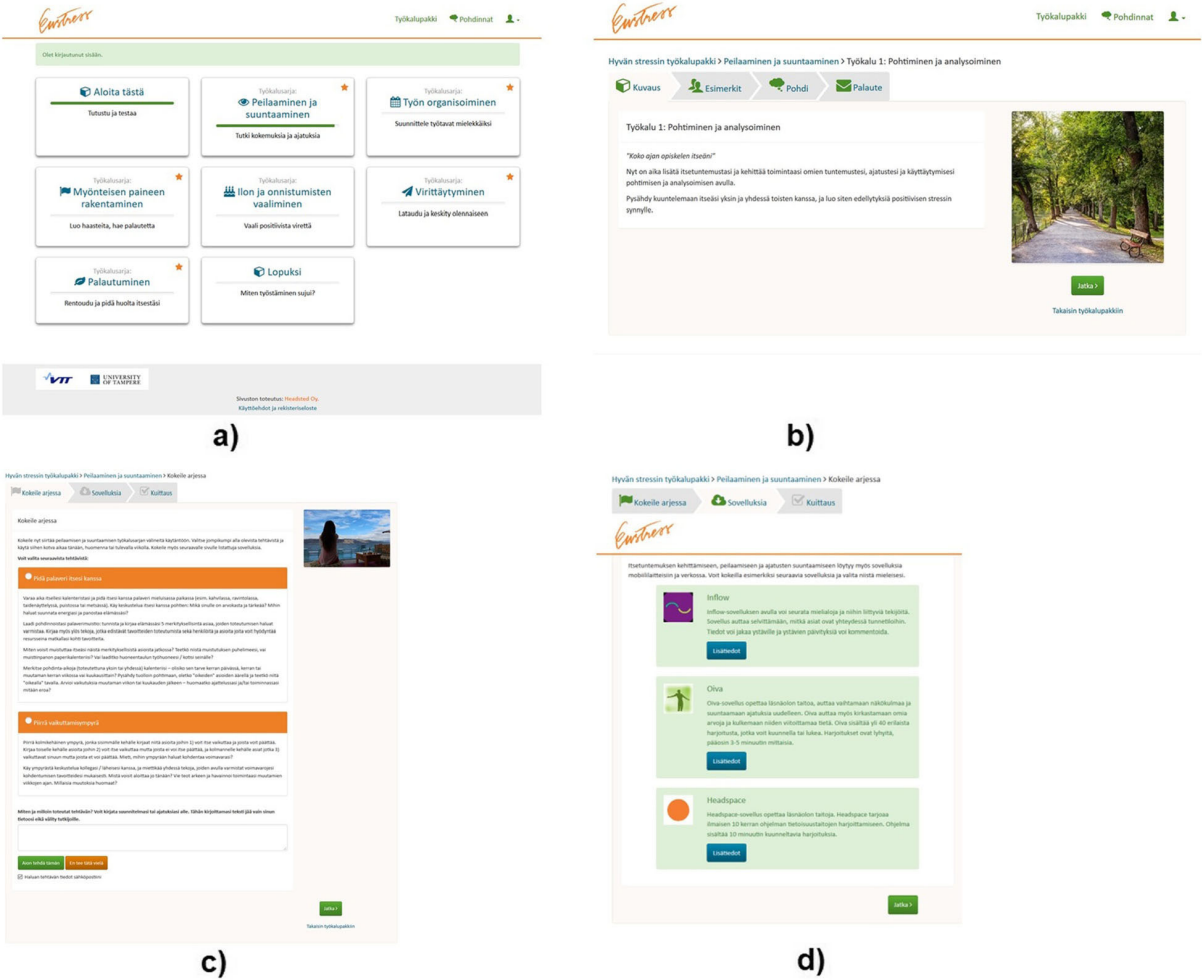
Screenshots illustrating the user interface of the Eustress Toolbox (in the original language): **a** main view with introductory module, 6 toolsets and an ending module; **b** structure of one tool, including tabs for introduction, entrepreneur quotes, reflection exercises and feedback; **c** off-line exercise; and **d** links to third-party applications

**Fig. 2:**
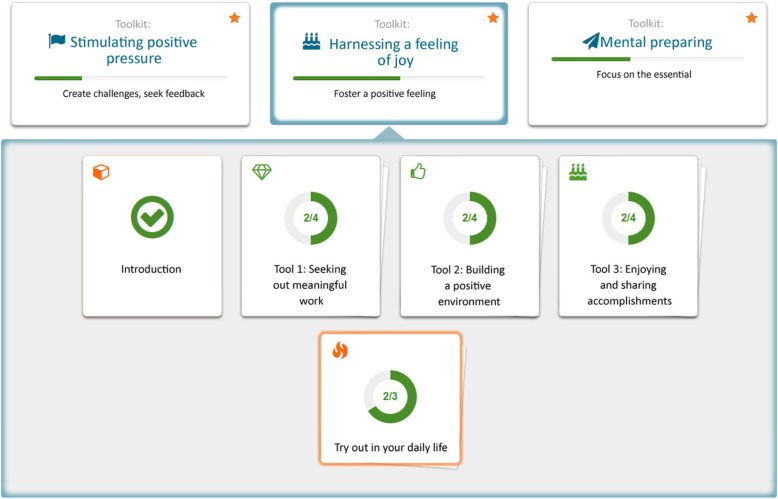
An example image of the Eustress Toolbox web service (translated to English). The structure of a toolset for harnessing a feeling of joy: an introduction, three tools and a section for trying out the tools in practice (including off-line exercises and 3rd party apps)

The toolbox consists of 6 toolsets (thematic areas) and altogether 24 tools (ways of thinking and working) for stimulating eustress (Table 1). When starting to use the toolbox, the user is instructed to go through an introductory module that contains information and instructions for using the service as well as a screening questionnaire for identifying the most useful toolsets for the user. The screening questionnaire consists of 21 statements, drawn from the previously collected qualitative data. The toolsets that are identified as the most useful for the user are marked with stars, to guide the user to focus on them in particular. Each toolset contains an introduction, 2–6 tools and 2–3 off-line exercises for practicing the skills in everyday life as well as recommendations of third-party applications that would support learning the skills (Figs. 1 and 2, see Additional file 1 for more screenshots of the service). After using the service for 3 weeks, an ending module is activated. The module contains a follow-up questionnaire, which repeats the screening questionnaire and visually summarizes the users’ progress related to the skills learnt in the toolbox.

**Table 1:** Toolsets and tools of the Eustress Toolbox

Toolset	Tools
Self-reflection and changing the mind-set	1) Self-reflection of thoughts, feelings and actions; 2) Shifting toward a positive point of view; 3) Gaining perspective; 4) Fostering trust in oneself and the future; 5) Regulating personal resources; 6) Sharing and sparring
Organizing work	1) Planning and scheduling; 2) Concretizing tasks and goals; 3) Working together; 4) Breaking routines
Stimulating positive pressure	1) Creating challenges; 2) Generating time pressure; 3) Seeking out challenging situations
Harnessing joy	1) Seeking out meaningful tasks; 2) Building a positive environment; 3) Enjoying and sharing success
Mental preparation	1) Focusing on the essential things; 2) Preparing for challenges
Recovery	1) Taking breaks; 2) Detaching from work; 3) Slowing the pace; 4) Releasing pressure; 5) Fostering physical and mental well-being; 6) Ensuring sufficient sleep

### Field study

Twenty-two participants volunteered in the field study, 13 of them were entrepreneurs and 9 were employees having an entrepreneur-type job description. Most participants (18/22) were female and their average age was 40.3 years (SD 7.2, range: 26–52 years). The average time in the current occupation was 4.8 years (SD 4.7, range: 0.1–20 years). The participants were all Finnish and they were working in different fields, for example, providing training and consulting, well-being services, legal services or software design.

The participants were recruited to the study through email lists of local entrepreneur associations and word of mouth. The participants were required either to work as entrepreneurs in small companies (being self-employed or having their own business) or to have an entrepreneur-like job description, characterized by high levels of independence and responsibility. After the participants expressed their interest, they were emailed a link to an online recruitment questionnaire, which was used to confirm their eligibility and to collect background information.

**Fig. 3:**
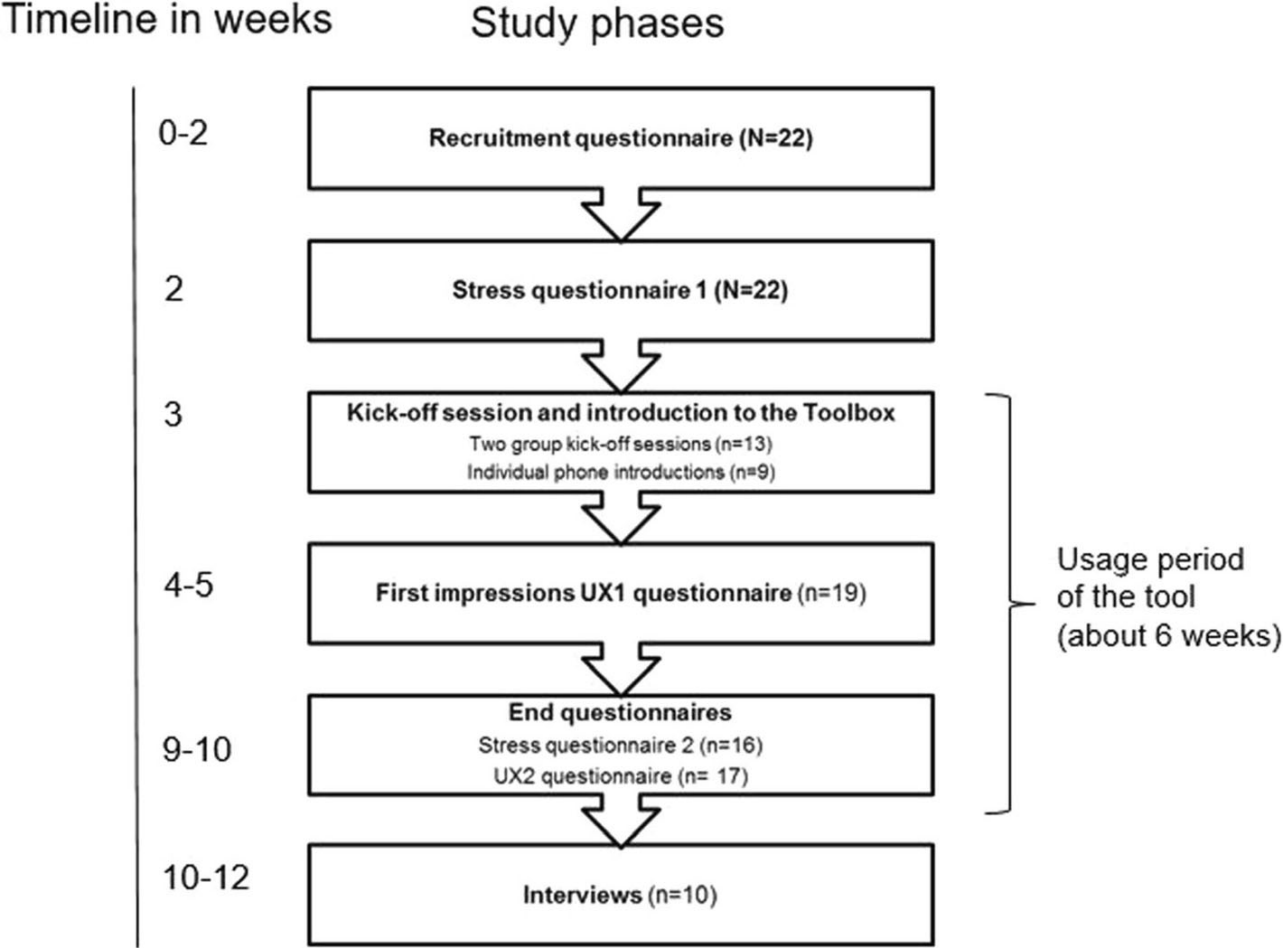
Study procedures

Figure 3 illustrates the study procedures. After filling in the recruitment questionnaire, the participants responded to an online questionnaire on stress and work engagement (Stress 1). Stress was measured with the Perceived Stress Scale (PSS) [6], which includes 10 items and measures psychological stress, i.e. how stressful people perceive the situations in their lives and how uncontrollable and overloaded people have perceived their life during the past month. Work engagement was measured instead of eustress because there are no validated scales for measuring eustress and because work engagement is closely linked to the experience of eustress. Work engagement was measured with the 9-item version of the Utrecht Work Engagement scale (UWES-9, [41]), which measures vigour, dedication and absorption. The UWES scale may be considered a three-dimensional or a one-dimensional scale, and thus an overall score or sub-scores of the tree dimensions may be analysed.

All participants were invited to one of two group kick-off sessions. The 1.5-h session introduced the phenomenon of positive stress, the study procedures and the Eustress Toolbox. If a participant was unable to attend the face-to-face session, an individual phone introduction session was scheduled, and an informed consent form and the other introductory material sent by email. All participants signed a written informed consent form, and those who attended the phone introduction session mailed the signed form back to the researchers. An ethical review was not deemed necessary based on the guidelines of the Ethics committee of the Tampere region (https://www.tuni.fi/en/research/responsible-research/ethical-reviews-in-human-sciences), where the study was conducted. The participants were reimbursed with movie tickets; the participants attending to a final interview received four movie tickets each and other participants two movie tickets.

The participants used the Eustress Toolbox independently, as a part of their daily life, during an approximately six-week period. The choice of six-week duration and the use of introductory kick-off sessions are in line with the systematic review of Heber et al. [21], which shows that short-to-medium web-based stress interventions have been more effective than over 9 weeks long and that guided interventions have been more efficacious. The researchers monitored when participants logged in to the service and if a user did not log in within 2 days of the kick-off session, a reminder was sent. The maximum number of reminders prompting the user to login for the first time was three.

After 1 to 2 weeks of use (mean 7.3 days), the participants were sent an online user experience questionnaire on their first impressions on the service. The questionnaire (UX 1; see Additional file 2) consisted of 17 questions or groups of questions. One group of questions contained 13 statements related to the user acceptance of the service based on the Technology Acceptance Model for Mobile Services (TAMM) [25]. TAMM model was selected as a basis, as it is broader than the initial Technology Acceptance Model [9], including the dimension of trust and measuring perceived value instead of perceived usefulness. In addition, questions about the content of the service and usage activity were included. Eleven of the questions were voluntary open questions on the first impressions, expectations and use of the service in the daily life.

After about 6 weeks of use (mean 41.5 days), the users were sent two end questionnaires: the same stress and work engagement questionnaire as at baseline (Stress 2) and a final user experience questionnaire (UX 2; see Additional file 3). UX 2 consisted of 37 questions or groups of questions. The 13 TAMM-based statements were repeated, with slight modifications for longer use. Also questions about ways of using the tools, usefulness of different features and toolsets, experienced benefits related to stress and learning new skills, and desired additional features were included. In addition, 17 voluntary open questions allowed participants to elaborate on the topics of the questionnaire.

Furthermore, 10 volunteer participants were interviewed to deepen the data obtained with the questionnaires. The interviews concentrated on the users’ prior knowledge on and experiences of positive stress, user experience with the Eustress Toolbox and the benefits and learning experiences gained from using the service (see Additional file 4). The interviews were semi-structured, lasted 60–90 min and were audio recorded.

The Eustress Toolbox also automatically collected log files on usage, which included all user actions in the service as well as feedback collected in the service. For the reflection exercises and other private notes made in the service, only the number of characters was stored in the log, not the content.

### Analysis

The PSS and UWES-9 questionnaires were scored according to their scoring manuals. An overall score for PSS was calculated, with possible scores ranging from 0 to 40. For UWES-9, both an overall score and sub-scale scores for vigour, dedication and absorption were calculated, with possible scores ranging from 0 to 6. The scores are reported as mean, standard deviation and minimum and maximum values. Changes in the scores from before to after using the service were analysed with paired comparisons using a Wilcoxon Signed Rank tests.

Log data were analysed to extract metrics for describing usage activity and ways of using the toolbox. We report usage metrics mainly for the six-week period after the first login. Usage metrics were calculated based on identified usage sessions. As the log file did not contain an explicit event marking the start or the end of a usage session, the rules for identifying a new session had to be created. To take into account possible breaks in usage sessions due to interruptions or completing off-line exercises, breaks of a maximum of 60 min were allowed, i.e. after a 60-min break, a new usage session was identified. Also other break lengths were tested, but based on visual inspection of the raw log file, 60 min seemed optimal. Completion of a tool was determined as having at least 5 events in a tool. Correlations between usage metrics and changes in stress ratings were studied using Spearman correlation.

Questionnaire data were analysed both quantitatively and qualitatively. The quantitative questions were analysed by calculating average scores of responses or numbers of participants responding in a certain way. For the TAMM-based questions, the average scores over the four TAMM dimensions (ease of adoption, ease of use, value and trust) were calculated. Scores of TAMM dimensions obtained from the UX1 and UX2 questionnaires were compared using the Wilcoxon Signed Rank test. Associations between perceived benefits related to positive and negative stress and usage metrics were studied using independent samples Mann-Whitney tests.

Only complete cases were analysed and reported in each type of quantitative data to avoid making assumptions on missing data. The statistical analyses were performed using the IBM SPSS Statistics Version 22 (IBM Corp, Armonk, NY, USA). Statistical significance threshold (α) was set at 0.05.

The qualitative questionnaire data and the interviews were analysed qualitatively by using the thematic coding method [15]. The coding was data-driven and done without reference to a preconceived framework. The codes were themed under the barriers of using the service, positive and negative user experience, perceived benefits, and development ideas proposed by the users. These themes were further analysed to find the key meanings and illustrative quotations.

## Results

In this section, we present the results of the study by answering the three research questions. The first part focuses on acceptance and user experience of the service. The second part presents results related to experienced benefits of using the service: changes in stress experiences and perceived benefits. Third, users’ development ideas and design implications are introduced.

The results will be presented based on the available data. The baseline questionnaire (Stress 1) and usage log files were available from all 22 participants. Nineteen participants responded to UX 1 questionnaire, 17 participants to UX 2, and 16 participants to the end questionnaire (Stress 2). Furthermore, 10 participants were interviewed.

### Acceptance and user experience of the service

All participants took the Eustress Toolbox into use. Eleven participants (50%) were reminded at least once to take the service into use. The mean duration between the kick-off session and the first login to the service was 4.2 (SD 4.6, min 0.5, max 19) days. The service was used, on average, on 3 days (mean 3.5, SD 1.8, min 1, max 7) and the usage spanned over a period of 25 days (SD 13, min 1, max 42). The mean duration of a usage session was 33 min (SD 26, min 5.4, max 123) and the total duration of use was 101 min (SD 59, min 29, max 246). On average, the participants used 3 toolsets (SD 1.9, min 1, max 6) and completed 38% of the tools in the service (SD 22, min 4.2, max 92). The most popular toolsets were the first three: Self-reflection and changing the mindset, Organizing work, and Stimulating positive pressure, with more than half of the participants using them. Based on the log data and the interviews, a typical way to use the service was to select a toolset recommended in the screening questionnaire and go through all the content and exercises before starting a new toolset.

According to the interviews and open data in the questionnaires, the most significant barrier to service use was participants forgetting to use it. Although a reminder features had been specified and implemented, due to a technical problem, the service did not send reminders during the field study period. The service was used rather occasionally, and thus it did not become a part of the users’ regular routines. Most of the users used the service only during their free time; only one third of them used it also during work days. Based on the interviews, the participants using the service during work days found it easier to find the time for using it than the participants who had used it only on their free time.

The acceptance of the service was studied with ratings of the service as a whole and its different aspects. Data from the interviews and the open data of the questionnaires gave additional information on the user acceptance.

**Fig. 4:**
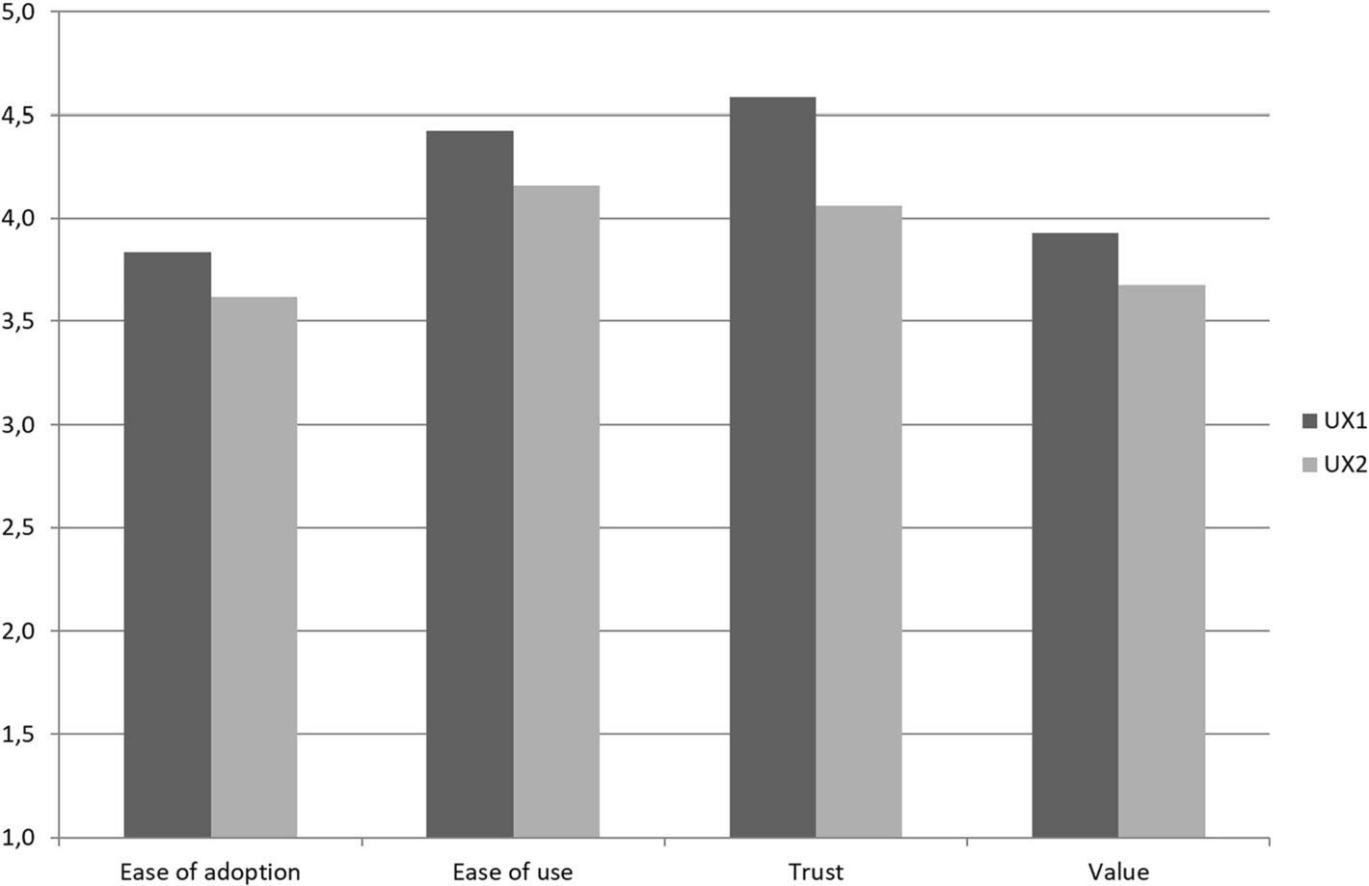
User acceptance ratings of the service from the first impressions questionnaire (UX 1) and the end questionnaire (UX 2)

The overall score given to the service on a scale from 0 to 10 was 7.5 (SD 1.1) in the UX 1 questionnaire and 7.2 (SD 1.3) in the UX 2 questionnaire. The likelihood to recommend the service was in line with the overall score: 7.7 (SD 1.7) in the UX 1 questionnaire and 7.5 (SD 1.5) in UX 2 questionnaire. The user acceptance ratings according to the TAMM dimensions are presented in Fig. 4. Even though the user ratings were generally rather positive, the acceptance of the service had slightly decreased during the usage period. However, a Wilcoxon Signed Rank test showed that the only statistically significant decrease was seen in the Trust dimension (Z = -2.81, *P* = 0.005). Further analysis into the individual statements revealed that decline was mainly due to a third statement added in the dimension in UX2, namely “The service has fulfilled my expectations,” which had an average score of 3.4.

**Fig. 5:**
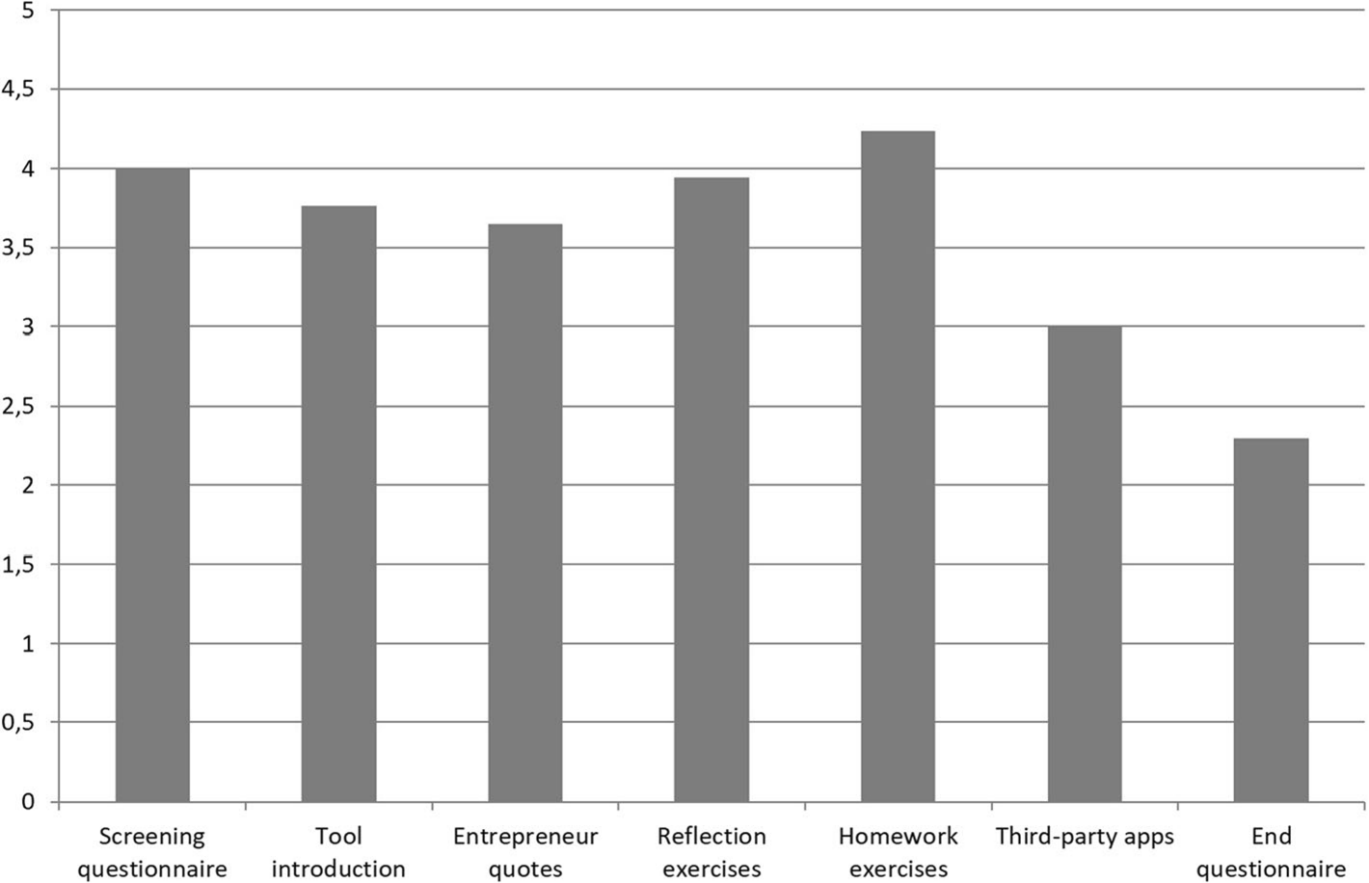
Usefulness of features, average ratings on a scale of 1 to 5

On a scale of 1 to 5, the participants rated off-line exercises (average rating: 4.2, SD 0.83), screening questionnaire (4.0, SD 1.2), and reflection exercises (3.9, SD 0.75) as the most useful features of the service (see Fig. 5). Quotes from other entrepreneurs were also perceived as useful. Sixteen out of 17 participants intended to continue using the service after the study period, and because of that, most of the users had not responded to the follow-up questionnaire (which shows the change in one’s eustress skills) of the service yet.

Off-line exercises were found to be especially useful, as they provided tools to integrate new skills into the daily life. They were commented to be small enough tasks, not requiring too big behavioural changes at a time, and to encourage the user to act – not just think about it.

I got a feeling of hope that with small actions I can get things going forward. It was good that they (the exercises) weren’t massive. Female, 50

Reflection exercises helped users identify their own practices and provided insights and inspiration on what the tools signified for them.

I first thought that I wouldn’t write down my thoughts as no-one else is going to read them. But when I did it, I realized that it makes you structure and understand and notice things better. It is not just some fuzzy stuff in your mind any more. Female, 34

The quotes from the entrepreneurs worked as a method to receive peer support and a way to obtain a new perspective or new ways of thinking.

I liked to read the comments from real people, how they experience things. They wake you up, that you know, someone thinks like this and it makes her behave like this. It actually is the best information that everyone doesn’t think the same way as you do. Male, 34

Organizing work (average rating: 3.3, SD 1.4) and Self-reflection and changing the mindset (3.3, SD 1.6) were rated as the most useful toolsets, which was also reflected in the usage data. In the interviews, self-reflection was experienced as an interesting and useful area that gave new viewpoints and compelled one to think about one’s values and choices. The Organizing work toolset was perceived as giving practical tools to manage one’s work, which are easy to adopt and have an impact on daily work.

According to the interviews, the content of the service was found to be interesting, but some considered the amount of information and exercises as overwhelming.

It guides you to go through pretty large entities. If you want to get through everything, it takes quite a lot of time. And you may forget what you did the last time. It makes you feel a bit overwhelmed. Male, 35

The recommendations based on the screening questionnaire helped in finding relevant themes, but tunnelling to more personalized content inside the themes was desired. Although more guidance for proceeding in the service was desired, some valued the possibility to freely search for the content relevant to oneself.

This left room for the right thing, the process that you need to go through yourself. It didn’t force you to do this and this to get forward. Female, 42

### Preliminary effects: changes in stress and perceived benefits

The participants’ perceived changes in stress were studied by two methods: by a validated perceived stress score (PSS) in the stress questionnaire before and after the study period, as well as by self-reported estimation in the UX2 questionnaire, with a further possibility to openly describe the experienced changes. Among the 16 participants who responded to the Stress 2 questionnaire, PSS scores decreased during the six-week study period (mean change − 4.4, SD 5.9). A Wilcoxon Signed Rank test showed that the participants’ stress score at the end was significantly lower than at the beginning (Z = -2.47, *P* = 0.013). Table 2 presents the PSS scores at the beginning and at the end of the study, and the changes calculated based on complete cases. The mean total UWES-9 score in the beginning was 4.9 (SD 0.82, 2.8–6.0), dedication sub-score was 4.9 (SD 1.1, 2.7–6.0), vigour sub-score was 4.7 (0.83, 2.7–6.0), and absorption sub-score was 5.0 (SD 0.78, 3.0–6.0). There were no significant changes during the study in any of the UWES-9 scores.

**Table 2:** Stress questionnaire scores as mean (SD, min-max)

Stress measure	Beginning	End	Change
PSS	15.1 (5.5, 6.0–22)	10.6 (4.2, 4.0–21)	−4.4 (5.9, −16–4) *

*P < 0.05, Wilcoxon Signed Rank test

The average percentage change in stress score was − 21.5% from the baseline score. Half of the participants who responded to the end stress questionnaire (8/16) had a clinically important change in their stress score, characterized by at least a 28% change in the PSS score, as defined by Eskildsen et al. [10]. There were no significant correlations between usage metrics (amount of use and completed tools) and the change in the stress score or the UWES-9 scores.

In the UX2 questionnaire, almost all (14/17) participants reported experiencing changes in stress during the study. Eleven participants reported changes in positive stress and 11 in negative stress. Sixteen participants reported having learnt new skills related to positive stress by using the service. The most commonly reported skill was recalling and celebrating successful work (14/17 participants).

Self-evaluated changes in positive stress were significantly associated with usage metrics. The participants who reported that their positive stress experiences increased had used significantly more toolsets (median number of toolsets used 5, IQR 3–6 vs. 1.5, IQR 1–2.3; Z = 2.86, *P* = 0.004) and completed more tools (median completion percentage 38%, IQR 33–67% vs. 13%, IQR 13–32%; Z = 2.83, *P* = 0.005) than those who did not report benefits. No associations were found between self-reported decreases in negative stress and usage metrics.

In the UX2 questionnaire, the participants gave different examples of the beneficial effects of the service:

By doing off-line exercises, I got to kick off a positive circle that things start going on and it gets you more enthusiastic. Female, 42

I have figured out new ways to get feelings of success at work. Female, 50

It has emphasized the importance of living in the moment. It has a great impact on stress and controlling it. Male, 34

The main benefits associated with the service usage were obtaining new insights and encouragement to try the skills in practice. For different participants the main insights were different: for example, finding new possibilities to have an impact on one’s work, noticing how one could foster living in the moment or realizing that in some cases procrastinating may be a feasible way of working. Some of the participants had adopted new practices: for instance, listing the most important tasks for the week on Monday morning or “having a meeting with oneself” (one of the off-line exercises) once a week while practicing aqua jogging.

### Development ideas and design implications

The participants proposed several design ideas; to overcome the current barriers, to integrate the service better into their life, and to make the service more engaging.

The most frequently stated improvement idea was the addition of reminders, which was requested by almost all participants (14/17) in the UX2 questionnaire and mentioned by all interviewees in the end interviews. Reminders were seen most useful if they had a direct link to the service and a concrete proposal for a new topic or exercise that might be useful for the user. Complementing usage with a physical handbook or a diary was also suggested. This might help in recalling the use of the service and support more versatile use contexts.

The service, in its current format, was not optimized for mobile use – using it when there is a convenient idle moment or a need for a more energetic or reassuring mind-set. The participants desired more instant access to the service and also a possibility to continue use from the previous state of the service. One participant compared the service to reading an e-book: the user should be guided back to the exact same spot that she last used. Some participants felt that opening the laptop and logging in required too much effort for a short use session. Instead, they wished the service was implemented as a stand-alone application, which would be easy to open.

More guidance was desired in proceeding inside the service. Desires were concretized as a personalized path, content appearing in the service in parts and a possibility to proceed in levels from beginner level to advanced use. In addition, the participants were missing clearer recognition of their achievements, as completing one toolset, for example, requires a considerable amount of time and effort. It was proposed that completing a toolset would be acknowledged by giving a summary of the content, proposing recommendations about what to do next or adding the toolset to a personal summary page of one’s progress.

Some of the proposed development ideas were related to the features that were currently liked in the service. The participants wanted more auditory exercises, which were considered to be more relaxing and effortless than reading text. As some of the exercises would be useful to try or repeat later, users hoped for a means to mark them as their favourites or as to-do tasks.

When the participants were asked to ideate what would make the service more attractive or even worth paying for, the addition of social or communal elements and expert feedback or coaching were proposed. The presence of other users or experts could make the use more humane and fun.

Even you researchers could have been more present in the service. You could see in the service that Pat (a researcher) has a bad day today and she wants to use this tool to cheer herself up. Or today we have a bit better buns with the coffee at the office. You would see some personalities, and it wouldn’t be just strict facts but something a bit more amusing. Female, 34

Also more advanced ways to analyse one’s own stress state – physiologically or with questionnaires – were desired.

Based on the user experience and users’ development ideas, the following key design implications were identified:

1. Make me put it into practice! - integrate into the daily hassle of entrepreneurs
  To make users utilize any service, it is crucial to support its everyday integration on practical level. This is especially important in the context of entrepreneurial work, as the daily work is often busy and fragmented. Regarding well-being information, it is typical to read it through, but often it does not lead into any changes in one’s behaviour. In our service, off-line and reflection exercises were perceived as useful as they link the content to the users’ life and supported applying it in the practice. Reminders and optimisation for mobile use would enhance the integration and enable more spontaneous usage. Another way to support integration is to offer versatile content for different usage situations. For instance, audio exercises are useful when reading is not an option. In addition, for some users, a physical or digital notebook or diary could further support practicing the new skills.
2. Guide me! - provide personal guidance while maintaining a possibility to explore
  Entrepreneurs may have very different needs regarding the service based on their prior knowledge, current work tasks and the work load, which may change frequently. For this reason, different users need different levels of guidance in using the service and the need may also vary between usage sessions. The Eustress Toolbox was designed to provide freedom to discover and empower the users to find the personally meaningful content. Even though the users valued the non-restriction of choice, they wanted more guidance in proceeding through the service. The design should offer guidance in a personally relevant way, for example, based on the screening questions or the achieved skills. In addition, the user’s state of mind could be taken into account by providing clearer guidance for achieving more energy or calmness, depending on the situation at hand.
3. Recognize me! - recognize the user’s progress and accomplishments in a meaningful way
  As the user puts time and effort into using the service, it is important to recognise the progress made walking through the material and rehearsing eustress skills. As entrepreneurs may lack feedback from a superior and colleagues, they may be especially motivated and delighted by the feedback given by the service. For example, completing a toolset should result in a meaningful recognition of the achievement, such as summarizing the content of the toolset and giving recommendations for additional content.
4. Show me what the others do! - support implicit learning from peer entrepreneurs
  Supporting learning from peers was one of the key design principles when designing the service. In the service, learning from peers was enabled by providing quotes from entrepreneurs who told about the ways of thinking and working that had helped them achieve eustress. The quotes were perceived as insightful and inspiring, while some participants also wished for actual interaction with peers to be included in the service. As entrepreneurs may lack support from colleagues and a feeling of belonging to a work community, a possibility to connect with peers or relate to their experiences is valuable.

## Discussion

Earlier studies have reported entrepreneurs’ special relationship with stress and underlined the importance of their stress management skills [3, 5, 39, 46]. This study investigated the feasibility of a new web service, the Eustress Toolbox, for stimulating the positive side of stress among entrepreneurs and people doing entrepreneur-like work. We aimed to study the user experience, user acceptance and preliminary effectiveness of the service, and identify the design implications that would make the service more attractive and integrate it better into entrepreneurial life.

### User experience, user acceptance and design implications

The overall user experience of the Eustress Toolbox was rather positive – the content was found to be interesting and almost all the users wanted to continue using the service after the field study period. The reflection and off-line exercises as well as the screening questionnaire (giving personal recommendations about toolsets) were perceived as useful, as they helped linking the content to the users’ life. Also the entrepreneur quotes were appreciated as they provided insights from the peer group and helped reflect on one’s thoughts.

The Eustress Toolbox scored the highest in the perceived ease of use and trust dimensions, where the average of responses exceeded 4 out of 5 in both questionnaires. Research on the original TAM model has shown perceived ease of use to be an important determinant of perceived usefulness and attitude towards use [43]. Trust is a new dimension, which has been included in the TAMM model to capture issues encountered when using mobile services, such as trusting the service provider and ensuring the privacy of the user [24]. Same concerns apply to health and wellness related services, making trust an important determinant of intention to use the service.

Despite the positive overall experiences, most users did not feel able to incorporate the service into their daily routines and the acceptance of the service had slightly decreased during the usage period. Creating routines and habits is key to engaging users over the long-term, as it has been shown that habit explains up to 40% of continued use of online shops (Gefen, 2011). The results showed that the Eustress Toolbox did not meet the expectations of the users. The Eustress Toolbox was used, on average, on 3.5 days, 33 min at a time, and 101 min in total during the six-week study period. The users used about three of the six toolsets and completed 38% of the tools in the service. Although the minimum amount of usage required to achieve effects is not yet known, at least weekly usage was expected, and therefore the usage frequency was lower than expected. This may have been affected by the lack of reminders (due to a technical problem) and a lack of optimization for mobile use in our prototype version. In addition, the limited total usage can partly stem from the goals and design of the toolbox: the Eustress Toolbox was designed for selective use letting the users choose the toolsets they feel they need to practice. The screening questionnaire supported this by recommending potentially useful toolsets for the users. Furthermore, some exercises were designed to be done off-line, and the time spent with these exercises is not recorded in the log data. Compared to other studies, the usage rates observed were relatively low. For example, Hasson et al. [19] reported a median of 48 logins during a 6-month intervention. Asplund et al. [2] reported participants completing an average of 5.83 out of 8 weekly modules. Van Straten et al. [47] reported 55% of participants completing the whole 4-module intervention. A pooled analysis of three randomized controlled trials of an internet-based stress management program showed that users completed 4.4 to 5.7 of the 7 available modules during an intervention that was planned to take 4–7 weeks [49].

To support service use becoming a habit, reminders would be helpful to users when starting to use the service, but later on, habit formation could be better encouraged by supporting trigger events [45], such as using the service when starting the work day. To maintain the user’s interest, it would also be useful to recognize the user’s efforts and accomplishments in the service and support usage in versatile contexts. Moreover, supporting social interaction could make the service more engaging.

Based on the user experience and users’ development ideas, four key design implications were identified. The implications are in line with the persuasive design principles of PSD Model [36], which was consulted already when designing the service. For example, providing quotes from peer entrepreneurs is one way to support social learning that is included also in PSD model, although the model describes it as learning from the other users. As the PSD model is wide and applicable in various contexts, prioritisation of the relevant features for a specific design may be difficult. The value of our design implications is to highlight the key issues that are relevant in the entrepreneurial context, to provide examples of implementing them and to present these issues in an intuitive way for HCI (human–computer interaction) practitioners. Each of the design implications supports the user in engaging with the system, which is especially important for facilitating experiences of positive stress, as the desire to achieve positive stress does not necessarily respond to a particular problem in one’s life but requires self-leadership.

Our results are consistent with earlier studies and design recommendations that identify integration into everyday life to be a common challenge in persuasive systems [1, 8]. Our results also reinforce the significance of learning from peers. Supporting learning from peers is a design principle that could be utilized more in designing persuasive systems and motivating users regarding the potential significance and effects of using a system. The implication has similarities to the design principle of open-ended social awareness as suggested by Baumer et al. [4], which refers to encouraging healthy decisions implicitly by increasing awareness of the health-related activities and decisions of others.

Although the design implications are based on using this particular service, they may benefit also design of other services targeted to enhance well-being at work and especially well-being of entrepreneurs and people having an entrepreneur-like job.

### Preliminary effects: changes in stress and perceived benefits

The participants’ negative stress decreased significantly during the study, as measured with the Perceived Stress Scale. The average change was − 21.5% from the baseline score. In their recent study, Eskildsen et al. [10] defined that a decrease of 28% is required in order to consider the change in PSS as clinically important. In our study, half of the participants responding to stress questionnaire 2 fulfilled this criterion. However, the participants in our study were not particularly stressed as their mean baseline PSS score of 15. corresponds to population averages of about 15 to 16 reported by Cohen and Janicki-Deverts [7], although it was slightly higher than entrepreneur average of 13.73 reported by Baron, Franklin and Hmieleski [3]. Thus, a large change in stress scores could not be expected. Also the UWES-9 score reflecting work engagement was at a high level, on average 4.9. In Finnish reference samples of altogether over 19,000 employees, the highest average work engagement scores of 4.83 were observed in senior managers [16]. We did not observe any changes in work engagement during the study, which is in line with the expectation that work engagement is a relatively stable state [42] and also with the study by Mauno et al. [32], where no changes in work engagement were seen during a 2-year follow-up of healthcare personnel.

More than half of the participants who responded to the second user experience questionnaire reported that they had experienced increases in positive stress and decreases in negative stress. The qualitative data may explain these evaluations; the users told that they had adopted new tools and practices especially for recognizing one’s thoughts and actions, for organizing work, and for recalling and celebrating success. A longer-term follow-up study would be needed to discover whether the new practices will stay in use and whether the users will actively continue stimulating their positive stress experiences. It was found that experienced increases in positive stress were significantly associated with more active use of the Toolbox, i.e. using more toolsets and completing a higher percentage of the tools. However, changes in negative stress (either self-reported or measured with PSS) were not associated with the usage activity of the Toolbox. This may indicate that the Toolbox actually was more successful at increasing experienced positive stress than decreasing negative stress. However, the study setting does not enable confirming this finding.

It is possible that some of the reported changes have occurred due to increased awareness of eustress and not only due to using the service. However, there was a significant association between self-evaluated changes in positive stress and Toolbox usage and, in the second user experience questionnaire, several users attributed the positive changes to the Toobox and specific exercises, which provides some confidence in the assumption that the changes were indeed brought about by the use of the Toolbox.

The results are in line with earlier intervention studies in the field of stress. Earlier research has shown positive outcomes in managing negative stress with web-based programs [2, 19, 47]. Our results support this, add to the understanding of entrepreneurs as a user group and provide preliminary evidence that approaching stress from a positive perspective can be feasible in targeting entrepreneurial stress. The results regarding the effectiveness of using the service are preliminary due to the limitations in the study setting, such as the sample size and the lack of a control group. However, we believe that reporting this preliminary feasibility is valuable due to the following differences in comparison to earlier papers: 1) our prototype service was preventive and targeted to entrepreneurs with fairly low baseline stress scores, 2) the service is based on entrepreneurs’ experiences and takes the entrepreneurial context into account, and 3) our results consider design implications alongside stress effects.

### Ethical aspects, limitations and future work

Approaching stress from a positive perspective does not come without ethical concerns. First, the line between positive and negative stress is thin: compulsive striving for positive stress or neglecting the need for recovery are likely to have negative consequences. Second, promoting positive stress in one’s life needs to be based on one’s intrinsic motivation. The use of the Eustress Toolbox could be enabled by the employer, but it cannot be used as a method to increase the external stressors or stressful work of employees. Third, fostering positive stress is intended to be used as a preventive approach – for people who are not suffering from intense negative stress. When experiencing intense negative stress, other stress management tools may be more useful than focusing on the positive aspects of stress.

The target group of this study was entrepreneurs and people having an entrepreneur-like job, which limits the generalizability of the results. However, new methods for finding suitable ways of working without forgetting well-being are relevant for a wider audience as well.

The study participants were selected on a voluntary basis, and thus the study sample included more females than males. Volunteer participants may not fully represent the whole target group. However, the process of fostering positive stress requires the user’s intrinsic motivation, which can be best achieved with a group of volunteers who are interested in mental well-being.

The study setting did not exclude the possibility that the participants’ stress had been decreased also because of other factors than the use of the Eustress Toolbox. Their stress experiences may have changed during the 6 weeks study period also because of the participation in the study as such, which may have made them think about their lifestyle and choices more - even if they had not used the Eustress Toolbox. Thus, the results regarding the effects on stress experiences should be considered as preliminary. Future studies could address this issue and repeat the study with a larger sample and with a control group. Furthermore, it would be interesting to test the acceptability and value of the improved service with other populations as well, such as employees, students or job seekers.

One limitation connected to the research setting is that there is no standardized measure for positive stress. Thus, the PSS scale for measuring negative stress and the qualitative reporting of stress experiences were used in this study. Developing a measure for positive stress would be an interesting research aim in the future.

In the future, an interesting holistic service could be one that would combine the means to enhance the stress balance in one’s daily life with physiological data on one’s bodily states. Our prototype of Eustress Toolbox could also be extended into a more comprehensive service concept with personal coaching, or it could be included as one module of a more general training in occupational well-being.

## Conclusions

This article presents the results of a six-week field study on the use of a web service for stimulating positive stress among entrepreneurs and people doing entrepreneur-like work. We studied the usage, user acceptance, user experience, and preliminary effects, combining the approaches of HCI and health interventions. The combination of the approaches proved to be fruitful: it revealed both the preliminary effects on stress and the user experience giving deeper understanding of the fit into the users’ life. In the Eustress Toolbox service, the users appreciated especially the off-line and reflection exercises, as well as the quotations from peers, but the design should have supported more active triggering to use the service. The following design implications were identified: *Integrate the service into the daily hassle of entrepreneurs, Provide personal guidance while maintaining a possibility to explore, Recognise the user’s progress and accomplishments in a meaningful way* and *Support implicit learning from peer entrepreneurs.* We hope that the findings give insights and inspiration for practitioners designing for well-being of entrepreneurs and encourage researchers to approach stress also from a positive perspective.

## Supplementary information

**Additional file 1.** Introduction of the Eustress Toolbox web service with screenshots. The screenshots are in the original language, but there are texts in English highlighting the parts of the service and the way the users are supposed to use the service.

**Additional file 2.** User experience questionnaire 1 developed for this study, translated to English.

**Additional file 3.** User experience questionnaire 2 developed for this study, translated to English.

**Additional file 4.** Theme interview questions developed for this study, translated to English.

## Data Availability

The datasets used and/or analysed during the current study are available from the corresponding author on reasonable request.

## Abbreviations

HCI: Human–computer interaction
PSS: Perceived Stress Scale
TAMM: Technology Acceptance Model for Mobile Services
UWES: Utrecht Work Engagement scale
UWES-9: 9-item version of the Utrecht Work Engagement scale
UX: User experience
