# Supplementary material for: Field study of a web service for stimulating the positive side of stress: entrepreneurs’ experiences and design implications
Source: BMC Med Inform Decis Mak. 2019 Oct 28;19:200. doi: 10.1186/s12911-019-0909-6 (PMC6816198; doi:10.1186/s12911-019-0909-6)
Supplement: Supplementary file 1 — Additional file 1. Introduction of the Eustress Toolbox web service with screenshots. The screenshots are in the original language, but there are texts in English highlighting the parts of the service and the way the users are supposed to use the service. [file 12911_2019_909_MOESM1_ESM.pdf]

User experience questionnaire 1: Eustress Toolbox service

This questionnaire concerns the usage of the Eustress Toolbox service (later ”service”). Responding to the questionnaire takes about 10 minutes. The purpose of the questionnaire is to find out your first impressions of the service. Please respond to the questionnaire as soon as possible.

The responses will be analyzed so that the identity of a respondent is not revealed.

Please respond to all questions. Thank you for your response!

1. **How many times have you used the service thus far?**
   ( ) None
   ( ) Once
   ( ) A couple of times
   ( ) More frequently

2. **What is your first impression of the service?**
   ____
   ____
   ____
   ____

3. **What were your expectations of the service? Have they been met so far?**
   ____
   ____
   ____
   ____

4. **Was the introduction to the usage of the service (in a face-to-face meeting or by phone) sufficient?**
   ( ) Yes
   ( ) No

5. **How did you find taking the service into use?**
   ____
   ____
   ____
   ____

6. **How have you started familiarizing yourself with the toolsets of the service?**

   ( ) I haven’t started yet
   ( ) I started from the toolset recommended by the service
   ( ) I started from the first toolset
   ( ) I started from a toolset that seemed interesting
   ( ) Otherwise, how? ____

7. **Have you integrated the service into your daily work and life? How?**
   ____
   ____
   ____
   ____

The questionnaires were originally sent as digital versions from an online tool, and thus the appearance of the questionnaire was different from this version. This is a translated version; the original questionnaire was in Finnish.

8. **Select the option that best describes your experience to each statement.**
(Likert scale from 1 to 5 (1=not at all useful to 5 = very useful, and 0 = can’t say)

It was easy to learn to use the service
The service use fits into my daily life
I find the service attractive
I enjoy using the service
The appearance of the service is pleasant
The content of the service is useful
The content of the service is interesting
The recommendations of the areas to focus on provided by the service seemed right to me
The service works without disturbance
I trust the information and content of the service
It is easy to use the service
It is easy to navigate within the service
The structure of the service is clear

You can comment on your response here.

____
____
____
____

9. **Have you found something interesting or useful from the web service thus far?**
____
____
____
____

10. **Has there been something useless or disturbing in the web service?**
____
____
____
____

11. **How do you find the content of the service thus far?**
(Likert scale, (Likert scale from 1 to 5 (1=totally disagree to 5 = totally agree, and 0 = can’t say)

The amount of content is suitable
The level of detail is suitable
The content is understandable
The content does not provide anything new
The content is presented in an interesting way

12. **Did the content meet your expectations? Should the content be different?**
____
____
____
____

The questionnaires were originally sent as digital versions from an online tool, and thus the appearance of the questionnaire was different from this version. This is a translated version; the original questionnaire was in Finnish.

13. **Have you searched for more information on the topics of the service?**
( ) Yes
( ) No

14. **If you have searched for more information, what kind of information and where?**
____
____
____
____

15. **How would you rate the service based on your usage thus far? Give an overall score on a scale from 0 to 10.**

____

16. **How likely would you recommend the service to others, like your friends or work community? Give an overall score on a scale from 0 to 10.**

____

To whom would you recommend the service? Why?
____
____
____
____

17. **Free thoughts: Frustrating or delightful experiences, development ideas, wishes for new content or something else?**
____
____
____
____
____
____
____
____

You will receive another questionnaire after the 6-week period.

Many thanks for your response!

The questionnaires were originally sent as digital versions from an online tool, and thus the appearance of the questionnaire was different from this version. This is a translated version; the original questionnaire was in Finnish.
