# Supplementary material for: Field study of a web service for stimulating the positive side of stress: entrepreneurs’ experiences and design implications
Source: BMC Med Inform Decis Mak. 2019 Oct 28;19:200. doi: 10.1186/s12911-019-0909-6 (PMC6816198; doi:10.1186/s12911-019-0909-6)
Supplement: Supplementary file 2 — Additional file 2. User experience questionnaire 1 developed for this study, translated to English. [file 12911_2019_909_MOESM2_ESM.pdf]

User experience questionnaire 2: Eustress Toolbox service

This questionnaire concerns the usage of the Eustress Toolbox service (later ”service”). Responding to the questionnaires takes about 15 minutes.

The responses will be analyzed in a way that the identity of a respondent is not revealed.

Please respond to all questions. Thank you for your response!

1. **How often have you used the service? Select the closest option.**
   ( ) Daily
   ( ) Several times a week
   ( ) Once a week
   ( ) Less often

2. **When did you typically use the service? You can select more than one option.**
   [ ] During the work day
   [ ] On free time
   [ ] Other, when? ________________________________

3. **Which device did you use for accessing the service? You can select more than one option.**
   [ ] Computer
   [ ] Tablet
   [ ] Phone
   [ ] I didn’t use the service

4. **Have you integrated service into your daily work and life? How?**
   ____
   ____
   ____
   ____

5. **How did you familiarize yourself with the toolsets of the service?**
   ( ) I familiarized myself with all the toolsets
   ( ) I familiarized myself with the toolsets recommended by the service
   ( ) I familiarized myself with the toolsets that seemed interesting
   ( ) I did not select certain toolsets but walked through the content from here and there
   ( ) Otherwise, how? ____

The questionnaires were originally sent as digital versions from an online tool, and thus the appearance of the questionnaire was different from this version. This is a translated version; the original questionnaire was in Finnish.

6. **Select an option that best describes your experience to each statement.**
(Likert scale from 1 to 5 (1=not at all useful to 5 = very useful, and 0 = can't say)

It was easy to learn to use the service
The service use fits into my daily life
I find the service attractive
I enjoy using the service
The appearance of the service is pleasant
The content of the service is useful
The content of the service is interesting
The recommendations of the areas to focus on provided by the service seemed right to me
The service works without disturbance
I trust the information and content of the service
It is easy to use the service
It is easy to navigate within the service
The structure of the service is clear

7. **You can comment on your previous response here.**
____
____
____

8. **Evaluate the usefulness of the following areas of the service:**
(Likert scale from 1 to 5 (1=not at all useful to 5 = very useful, and 0 = I haven't used the toolset)

Screening questionnaire
Introductions to the toolsets
Examples of the life of the entrepreneurs (quotes)
Reflection exercises
Off-line exercises
Follow-up questionnaire

9. **Evaluate the usefulness of the following toolsets (themes) of the service:**
(Likert scale from 1 to 5 (1=not at all useful to 5 = very useful, and 0 = I haven't used the toolset)

Self-reflection and changing the mindset
Organizing work
Stimulating positive pressure
Harnessing joy
Mental preparation
Recovery

10. **Have you found something interesting or useful in the web service thus far? What?**
____
____
____
____

The questionnaires were originally sent as digital versions from an online tool, and thus the appearance of the questionnaire was different from this version. This is a translated version; the original questionnaire was in Finnish.

11. **Has there been something useless or disturbing in the web service? What?**
    ____
    ____
    ____
    ____

12. **How did you find the content of the service?**
    (Likert scale, (Likert scale from 1 to 5 (1=totally disagree 5 = totally agree, and 0 = can't say)

    The amount of content is suitable
    The level of detail is suitable
    The content is understandable
    The content does not provide anything new
    The content is presented in an interesting way

13. **Did the content of the service meet your expectations? Should the content be different?**
    ____
    ____
    ____
    ____

14. **If you have searched for more information related to the content of the service, what information have you searched for and where? Why did you want to know more?**
    ____
    ____
    ____
    ____

15. **Did you do the reflection exercises of the service? Select the best option for you.**
    ( ) No, I didn't
    ( ) Yes, a couple of exercises
    ( ) Yes, several exercises

16. **You can comment on the reflection exercises here.**
    ____
    ____
    ____
    ____

17. **Did you do the off-line exercises of the service? Select the best option for you.**
    ( ) No, I didn't
    ( ) Yes, I tried out one exercise
    ( ) Yes, several exercises

The questionnaires were originally sent as digital versions from an online tool, and thus the appearance of the questionnaire was different from this version. This is a translated version; the original questionnaire was in Finnish.

18. You can comment on the off-line exercises here.

____
____
____
____

19. Did you try out 3rd party applications that were introduced in the service?

( ) Yes
( ) No

20. Which applications have you tried out?

____
____
____
____

21. You can comment on the 3rd party applications here.

____
____
____
____

22. **Have you used other applications or tools for stress management, recovery or eustress skills? Which applications and for what purpose?**

____
____
____
____

23. Do you think that using the service has had an impact on your experiences of positive stress?

( ) No, it hasn't
( ) Yes, somewhat
( ) Yes, significantly

24. Do you think that using the service has had an impact on your experiences of negative stress?

( ) No, it hasn't
( ) Yes, somewhat
( ) Yes, significantly

25. If you think that the service has had an impact on your stress experiences, what kind of an impact? How have you noticed that?

____
____
____
____

The questionnaires were originally sent as digital versions from an online tool, and thus the appearance of the questionnaire was different from this version. This is a translated version; the original questionnaire was in Finnish.

26. Have you learned following skills by using the service?
(Likert scale, (Likert scale from 1 to 5 (1=not at all to 5 = very much, and 0 = can't say)

Recognising my own thoughts and experiences
Managing work
Creating challenges
Recalling and celebrating successful work
Preparing to challenging situations
Detaching from work

27. Have you learned something else by using the service? What?
____
____
____
____

28. Which additional services would you like to use as support? Select up to 5 options
[ ] Online conversations with other users
[ ] Meetings with other users
[ ] Meetings with an expert
[ ] A possibility to contact an expert online
[ ] Feedback on progress
[ ] Gamified features (like collection of points)
[ ] Physiological measurements of stress
[ ] Wider possibilities for self-assessment (tests)
[ ] Co-use within work community
[ ] Other, what? _______________________________

29. Which target group would you think would get most value from the service?
____
____
____
____

30. Would you be ready to pay for this service?
( ) Yes, as it is ( ) Yes, if it offered more content
( ) Yes, if it offered regularly new content
( ) Yes, if it was a part of a wider eustress service
( ) Yes, if ______________________________________
( ) No, I wouldn't

31. How much would you pay for this service (current version)?
You can select a monthly or a one-time payment according to your preference.
_______e / month or
_______e as a one-time payment

32. You can comment on your previous response here.
____
____
____
____

The questionnaires were originally sent as digital versions from an online tool, and thus the appearance of the questionnaire was different from this version. This is a translated version; the original questionnaire was in Finnish.

33. How would you rate the service based on your usage? Give an overall score on a scale from 0 to 10.

____

34. How likely would you recommend the service to others, like your friends or work community? Give an overall score on a scale from 0 to 10.

____

35. **Are you going to use the service after the study period?**
( ) Yes
( ) No

36. You can comment on your previous response here.
____
____
____
____

37. **Free thoughts: Frustrating or delightful experiences, development ideas, wishes for new content or something else?**
____
____
____
____
____
____
____
____

Many thanks for your response!

The questionnaires were originally sent as digital versions from an online tool, and thus the appearance of the questionnaire was different from this version. This is a translated version; the original questionnaire was in Finnish.
