# Supplementary material for: Field study of a web service for stimulating the positive side of stress: entrepreneurs’ experiences and design implications
Source: BMC Med Inform Decis Mak. 2019 Oct 28;19:200. doi: 10.1186/s12911-019-0909-6 (PMC6816198; doi:10.1186/s12911-019-0909-6)
Supplement: Supplementary file 3 — Additional file 3. User experience questionnaire 2 developed for this study, translated to English. [file 12911_2019_909_MOESM3_ESM.pdf]

Theme interview for the participants of the study

1. **Background**
   a) Could you tell shortly about you and your business?
   b) What is your daily work like?
   c) How was your work and life during the piloting of the service (work, perceived stress, special events, family etc)?

2. **Eustress phenomenon**
   a) When you started the research process, how familiar were you with the phenomenon of positive stress?
   b) How often / when do you recognize a feeling of positive stress?
   c) What kinds of factors facilitate your experience of positive stress?
   d) Can you give an example of a recent situation at work when you have felt positive stress?
   e) Do you think that this perspective (the positive side of stress) is relevant for you or would it be better to concentrate on minimizing negative stress only?

3. **Usage of Eustress web service**

   a) Background: Have you used well-being related web services or other digital solutions before?

   b) How did you use the Eustress service?

   I. When did you use it (what time / in what kind of situations / mental state)?
   II. Where did you use it? Which device did you use for accessing the service?
   III. Did you have specific routines or ways of using the service?
   IV. Did you use the service by yourself or with others?
   V. Did you talk about the service with others? With whom? Did you want to share something?

   c) Can you recall a good and/or a bad usage experience? Could you tell about them?
      I. What kind of feelings did you have?

   d) Which part(s) of the service did you like most?
      i. Which topics did you find useful? Why?
      ii. Which tasks were the most useful for you? Why?

   e) Which part(s) of the service did you not like? What did not work for you? Why?

   f) What was missing from the service?

   g) Did you try out the mobile applications provided in the service? What did you think about them?

   h) Did using the service become a part of your daily life? Why / why not? How could it integrate better to your work and life?

   i) What kind of development ideas do you have?

This is a translated version; the original interview was in Finnish.

4. **Learning of skills and perceived benefits**
   a) Do you think that you have learned something new from using the web service? What have you learned?
   b) Have you learned something about yourself and your experiences of stress?
      i. Has your impression and understanding of the phenomenon of positive stress changed during the trial? How?
   c) Did the usage have some impact to you or your daily life? What kind of impact?
      i. Impact on your work / work day / work community?
   d) What factors facilitated (e.g. increased, deepened or speeded up) or hindered learning new skills?
   e) Did you have expectations that were not met, e.g. skills that you would have liked to gain but you did not (at least during this usage period)?

5. **Free comments**
   a) Would you like to add something or tell more about your experiences with the service?
