# Supplementary material for: Field study of a web service for stimulating the positive side of stress: entrepreneurs’ experiences and design implications
Source: BMC Med Inform Decis Mak. 2019 Oct 28;19:200. doi: 10.1186/s12911-019-0909-6 (PMC6816198; doi:10.1186/s12911-019-0909-6)
Supplement: Supplementary file 4 — Additional file 4. Theme interview questions developed for this study, translated to English. [file 12911_2019_909_MOESM4_ESM.pdf]

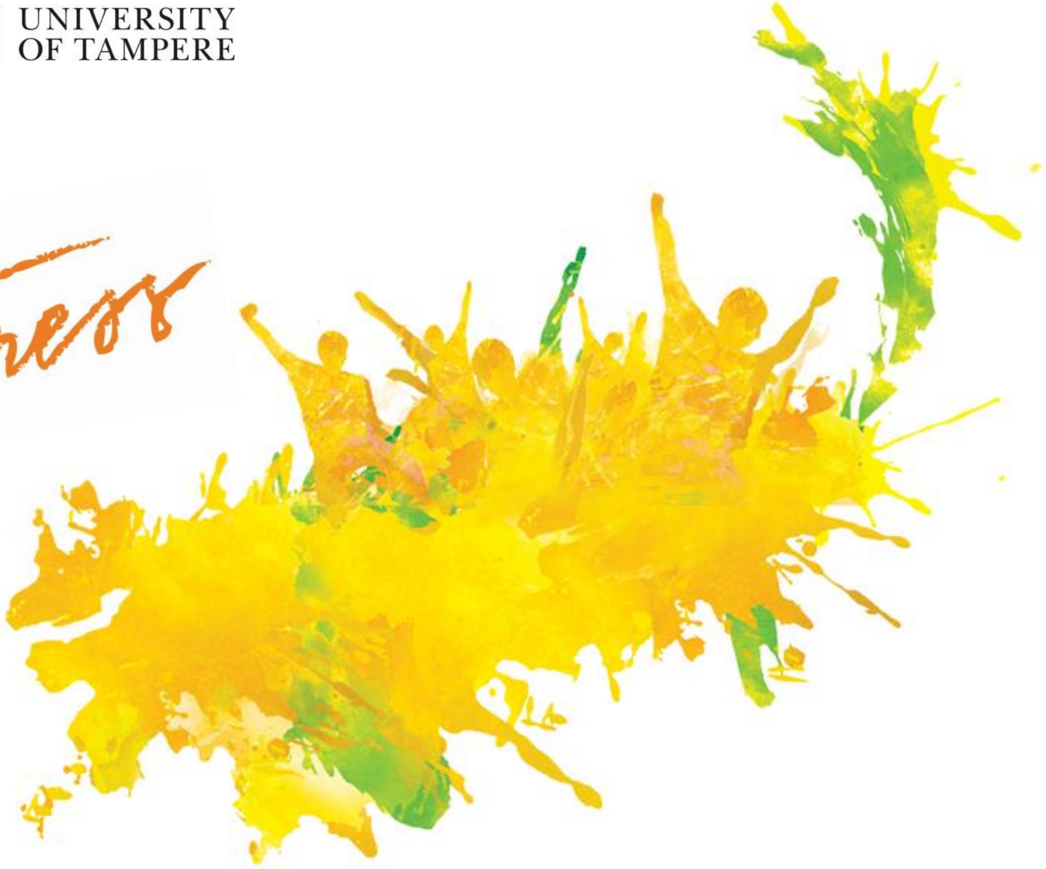

Eustress tool
Introduction of the web service

Päivi Heikkilä, Elina Mattila and Mari Ainasoja

Taking into use (1/2)

Web address: hyvastressi.fi

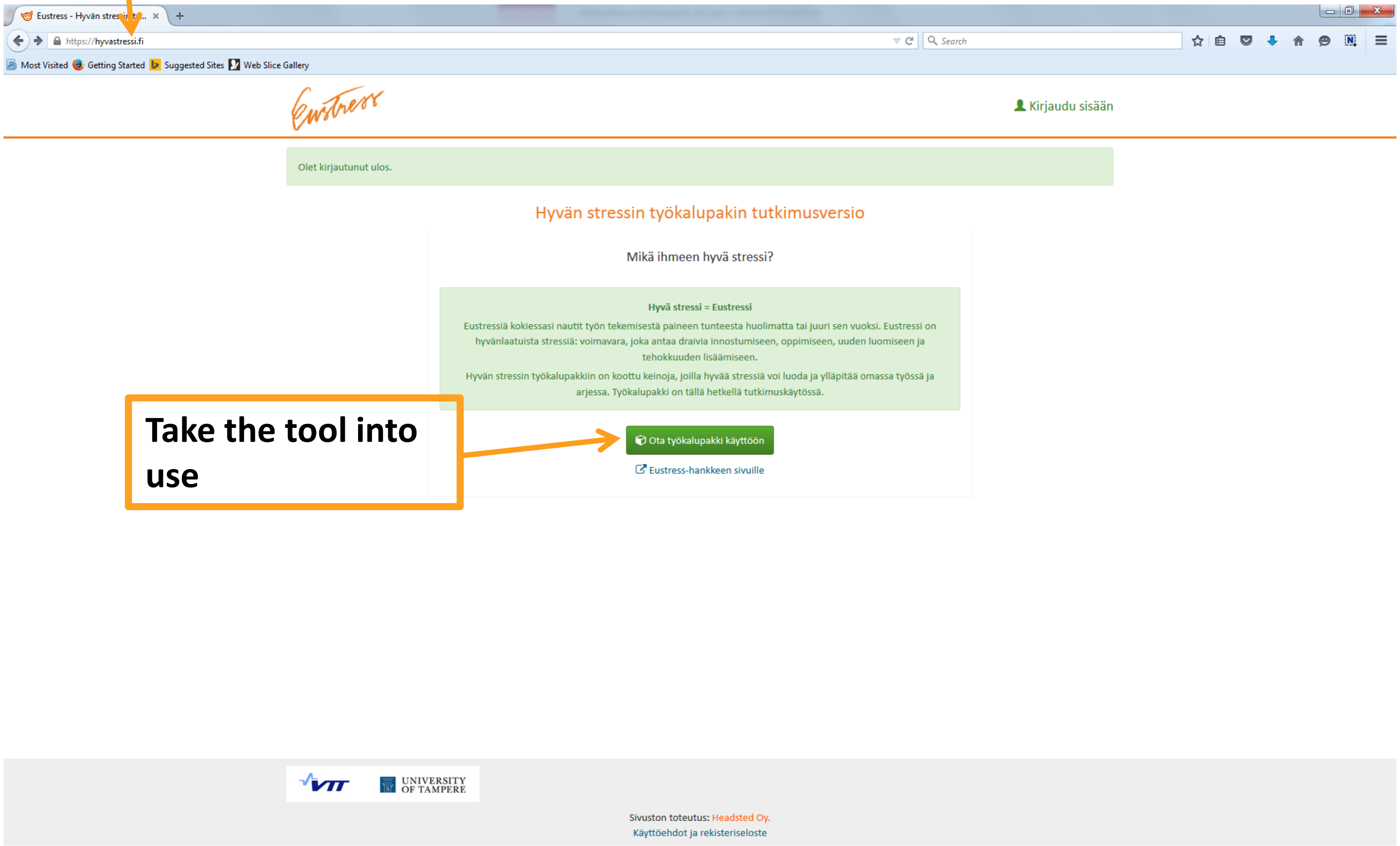

Take the tool into use

Taking into use (2/2)

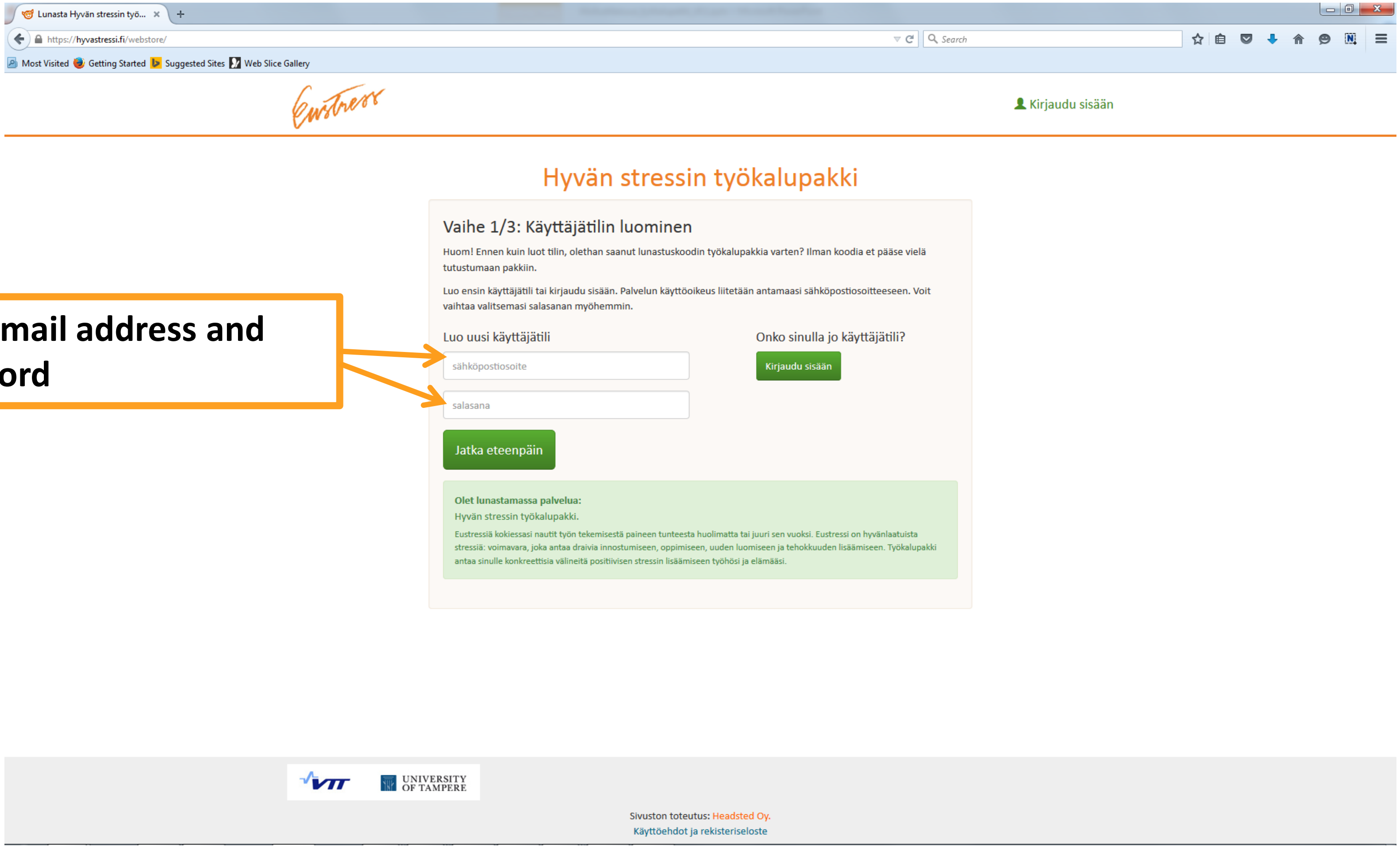

Your email address and password

Login

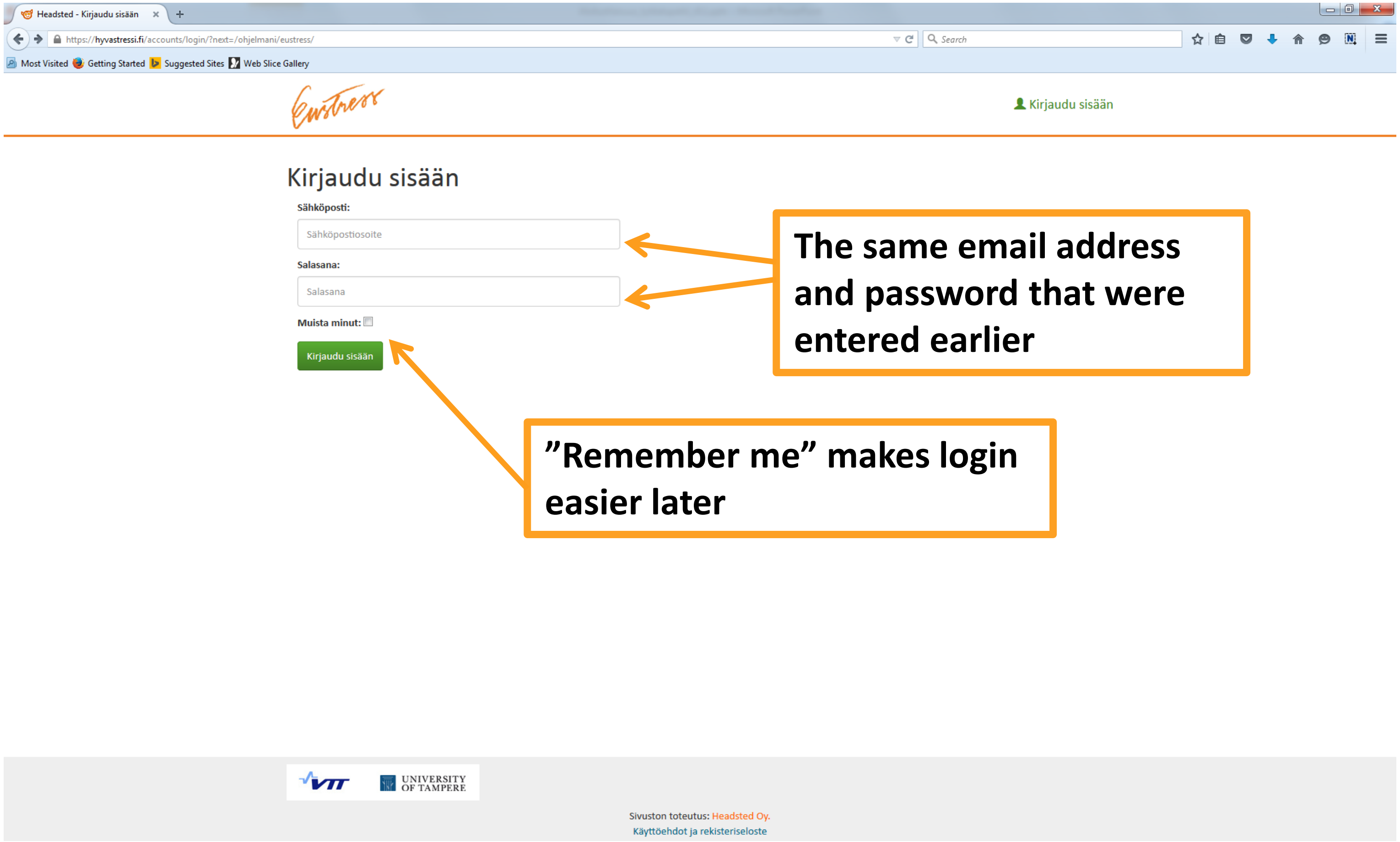

The same email address and password that were entered earlier

”Remember me” makes login easier later

Starting to use the service

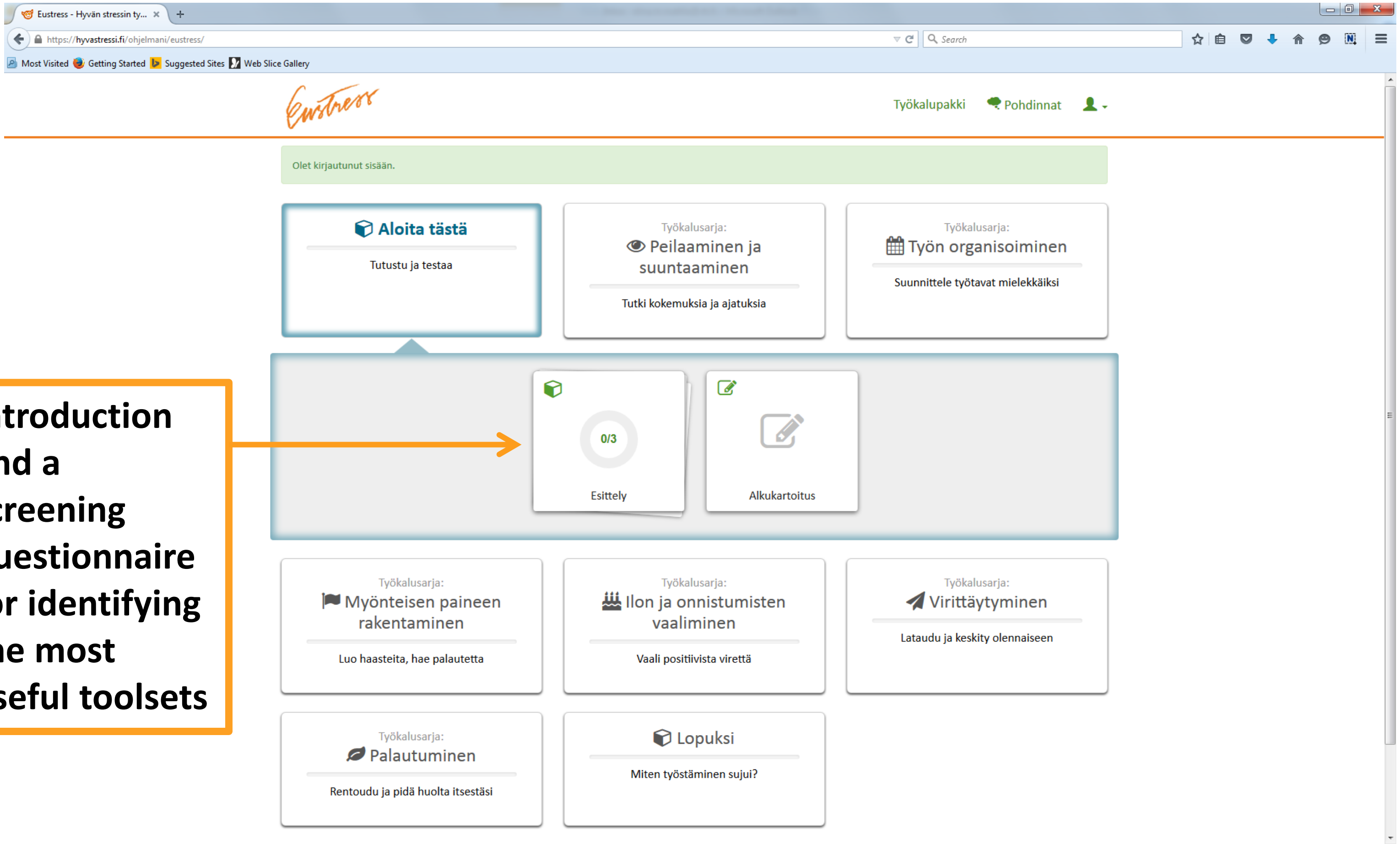

Introduction and a screening questionnaire for identifying the most useful toolsets

Screening questionnaire

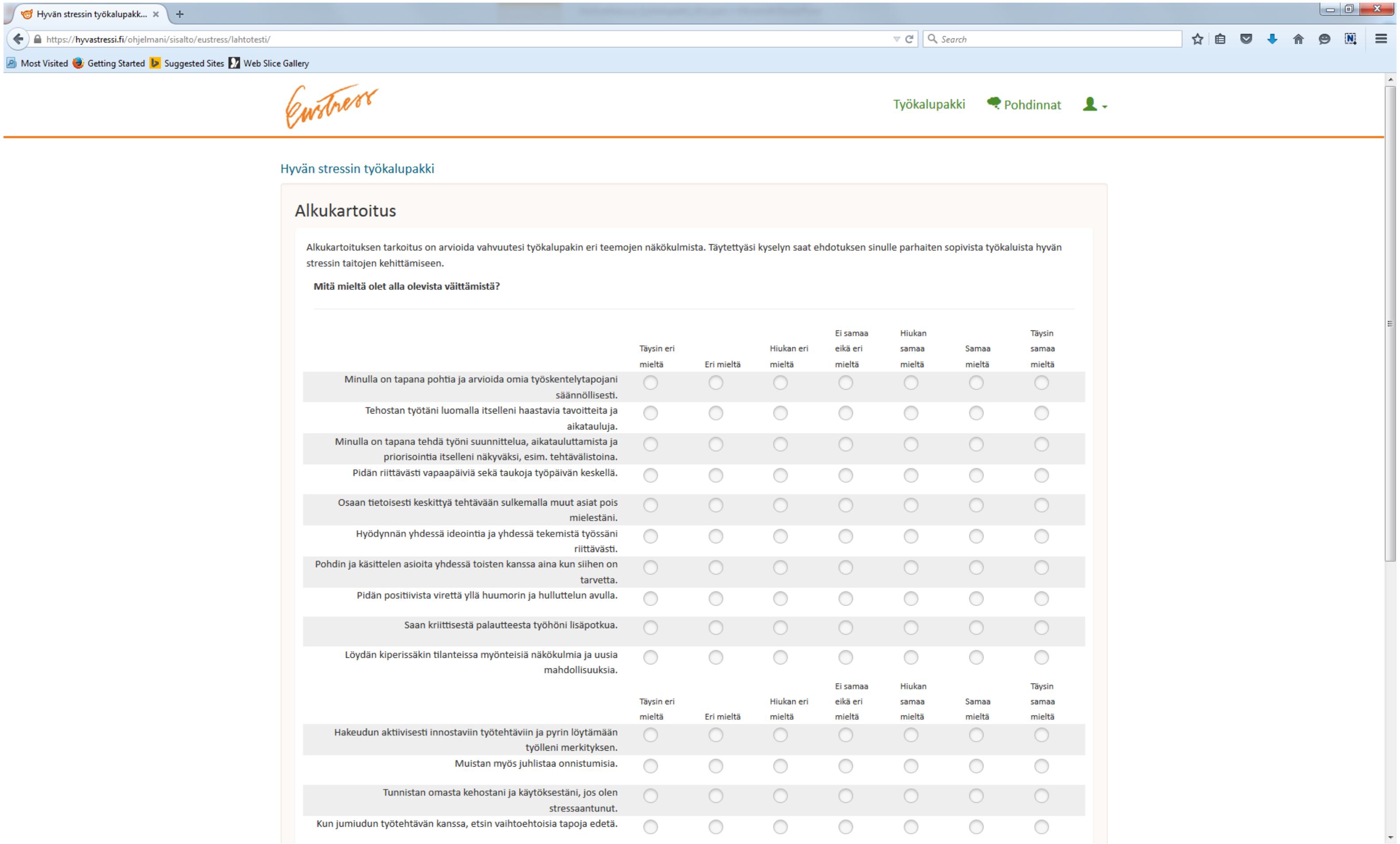

Hyvän stressin työkalupakk... ×

https://hyvastressi.fi/ohjelmani/sisalto/eustress/lahtotesti/

Most Visited | Getting Started | Suggested Sites | Web Slice Gallery

Työkalupakki | Pohdinnat

Hyvän stressin työkalupakki

Alkukartoitus

Alkukartoituksen tarkoitus on arvioida vahvuutesi työkalupakin eri teemojen näkökulmista. Täytettyäsi kyselyn saat ehdotuksen sinulle parhaiten sopivista työkaluista hyvän stressin taitojen kehittämiseen.

Mitä mieltä olet alla olevista väittämistä?

	Täysin eri mieltä	Eri mieltä	Hiukan eri mieltä	Ei samaa eikä eri mieltä	Hiukan samaa mieltä	Samaa mieltä	Täysin samaa mieltä
Minulla on tapana pohtia ja arvioida omia työskentelytapojani säännöllisesti.							
Tehostan työtäni luomalla itselleni haastavia tavoitteita ja aikatauluja.							
Minulla on tapana tehdä työni suunnittelua, aikatauluttamista ja priorisointia itselleni näkyväksi, esim. tehtävälistoina.							
Pidän riittävästi vapaapäiviä sekä taukoja työpäivän keskellä.							
Osaan tietoisesti keskittyä tehtävään sulkemalla muut asiat pois mielestäni.							
Hyödynnän yhdessä ideointia ja yhdessä tekemistä työssäni riittävästi.							
Pohdin ja käsittelen asioita yhdessä toisten kanssa aina kun siihen on tarvetta.							
Pidän positiivista virettä yllä huumorin ja hulluttelun avulla.							
Saan kriittisestä palautteesta työhöni lisäpotkua.							
Löydän kiperissäkin tilanteissa myönteisiä näkökulmia ja uusia mahdollisuuksia.							

	Täysin eri mieltä	Eri mieltä	Hiukan eri mieltä	Ei samaa eikä eri mieltä	Hiukan samaa mieltä	Samaa mieltä	Täysin samaa mieltä
Hakeudun aktiivisesti innostaviin työtehtäviin ja pyrin löytämään työlleni merkityksen.							
Muistan myös juhlistaa onnistumisia.							
Tunnistan omasta kehostani ja käytöksestäni, jos olen stressaantunut.							
Kun jumiudun työtehtävän kanssa, etsin vaihtoehtoisia tapoja edetä.							

Feedback from the screening questionnaire

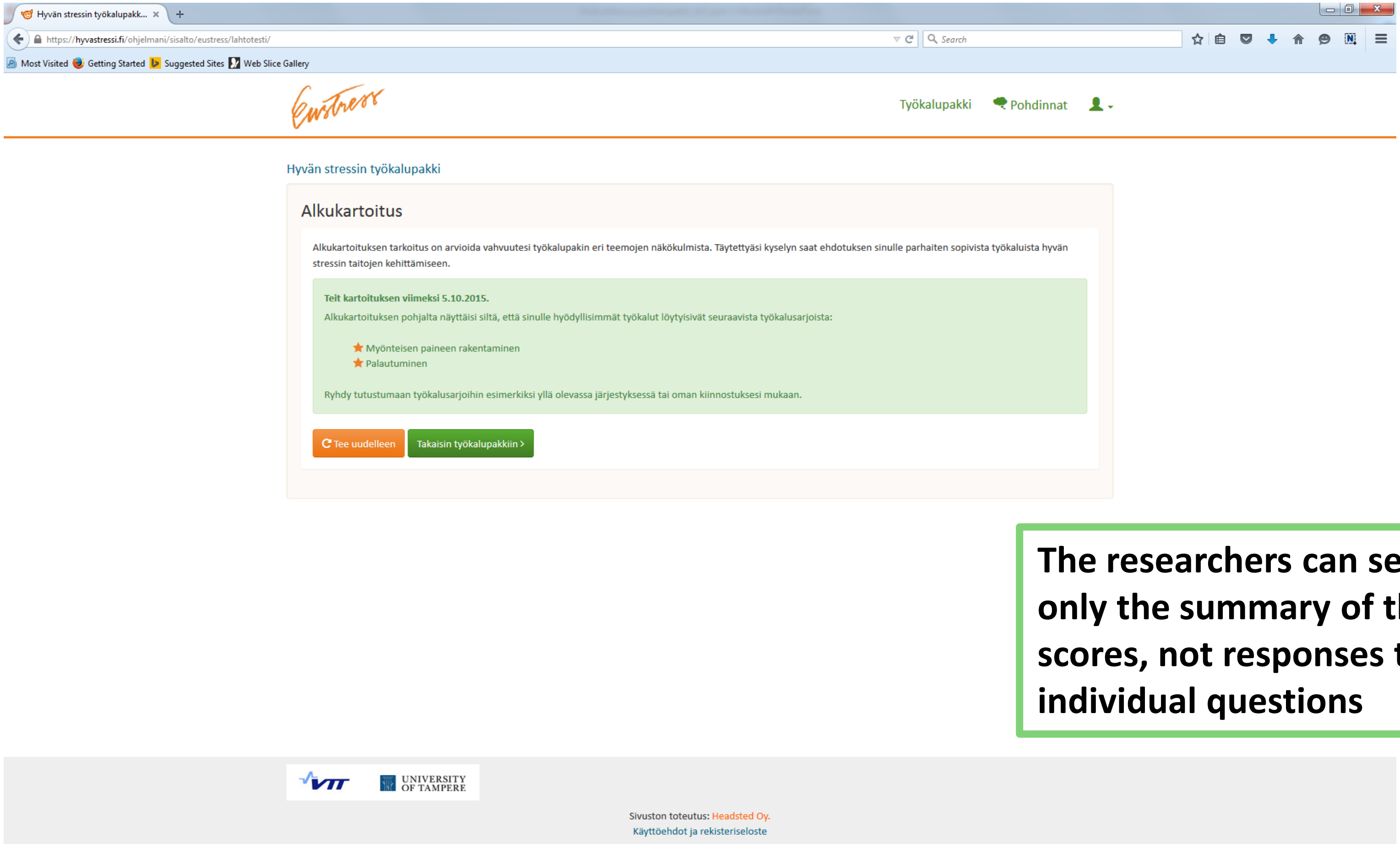

The researchers can see only the summary of the scores, not responses to the individual questions

Structure of the service (1/2)

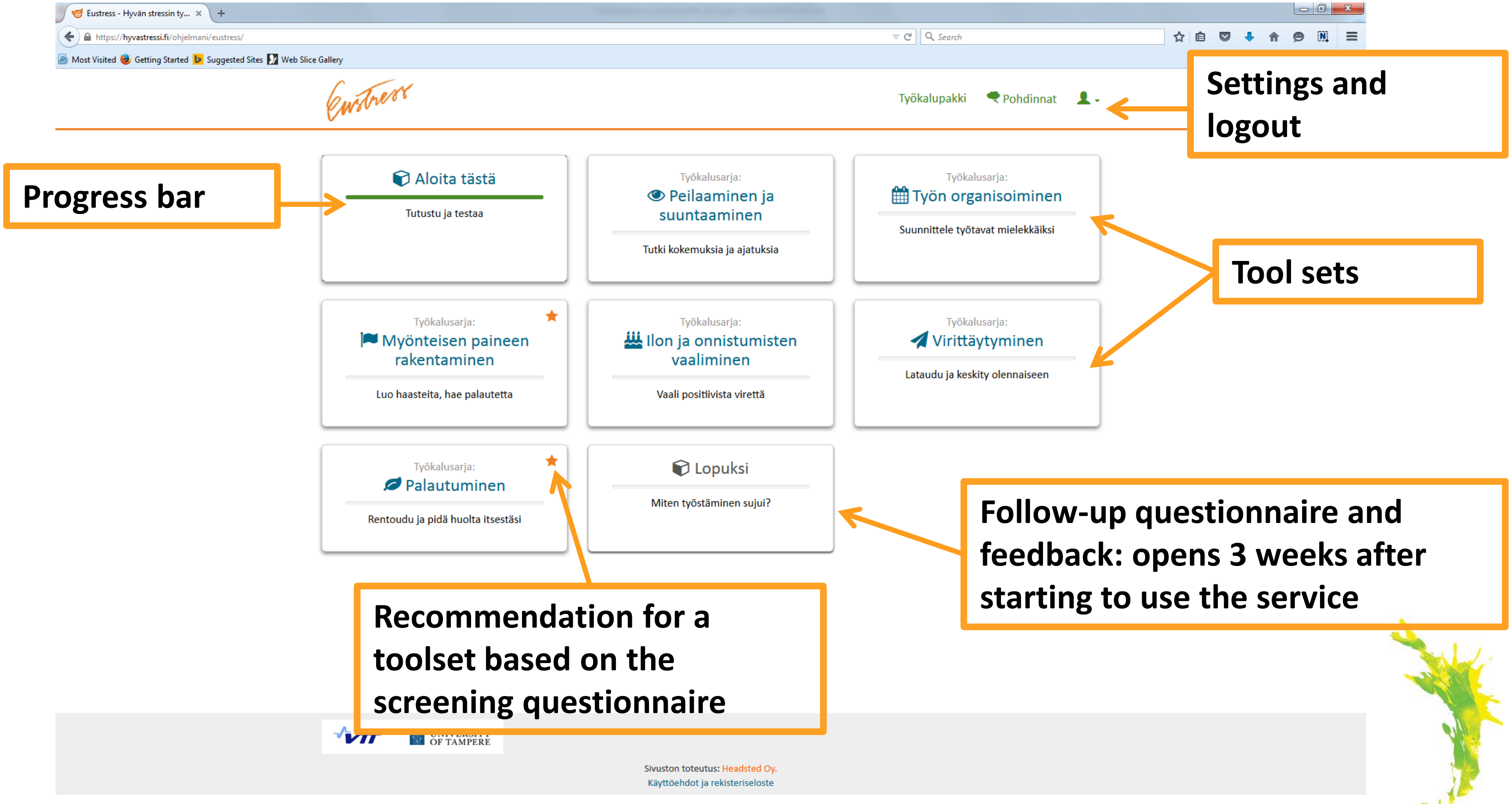

Eustress

Structure of the service (2/2)

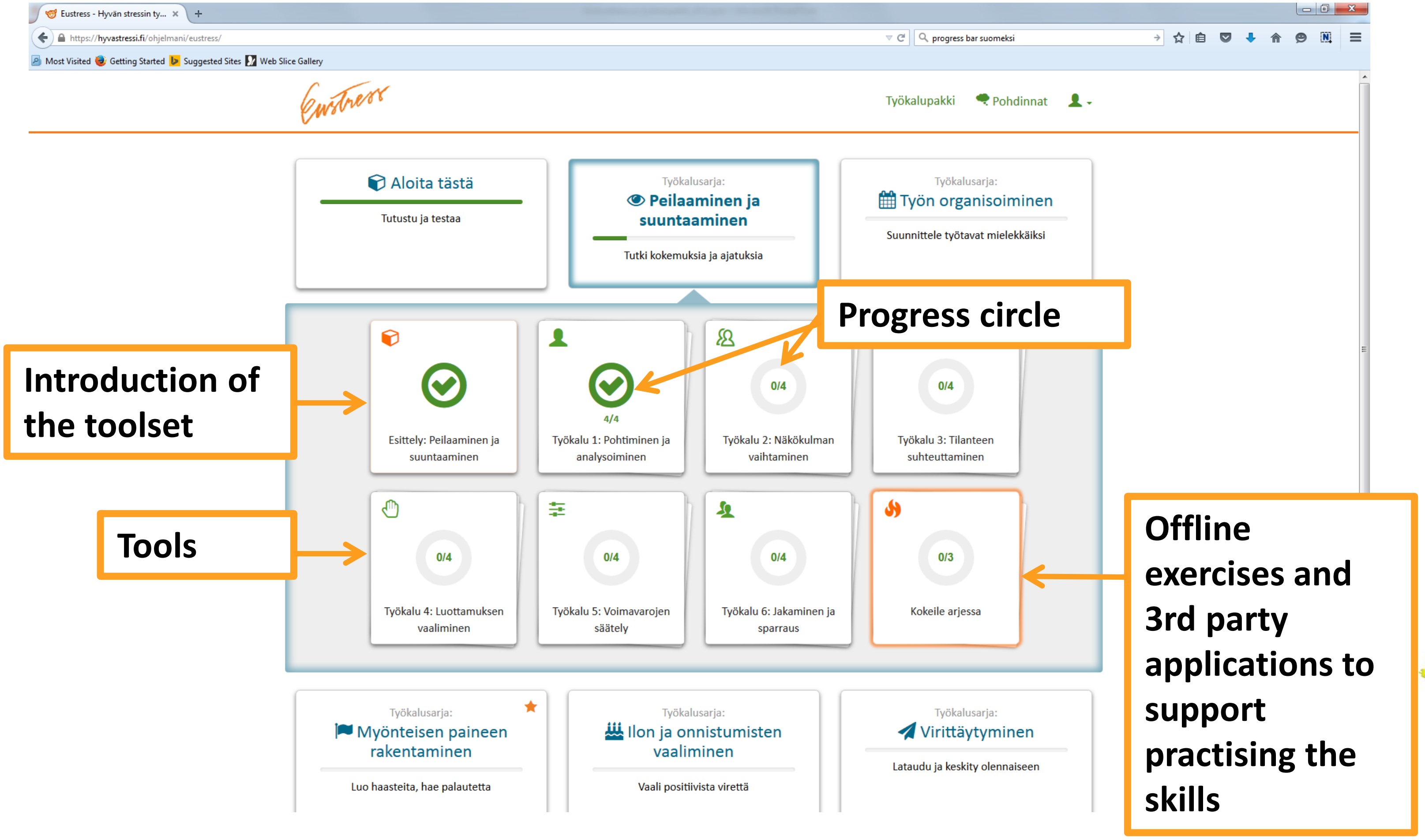

Content of a tool (1/4): Description

Proceeding: two options

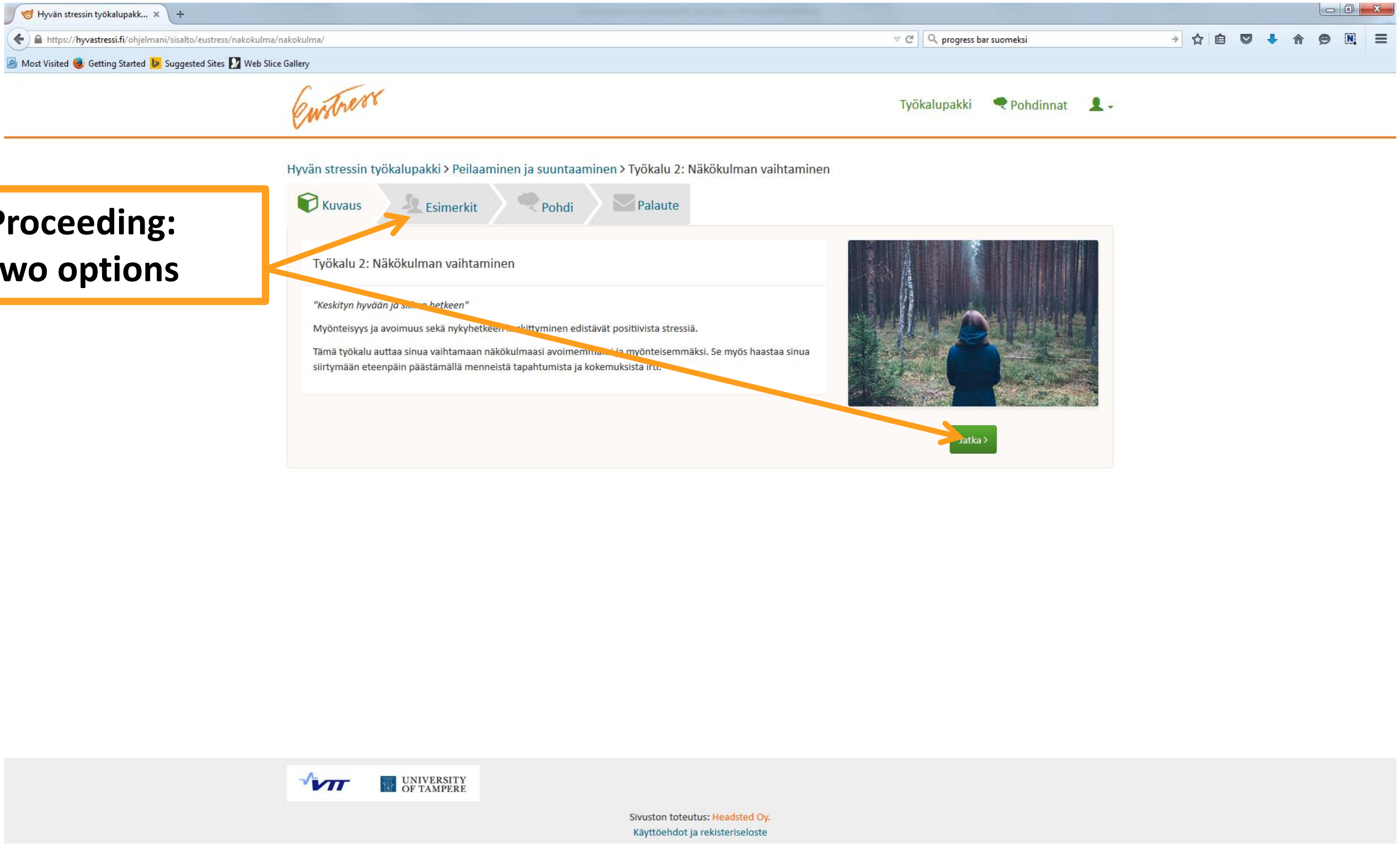

Content of a tool (2/4): Example quotes

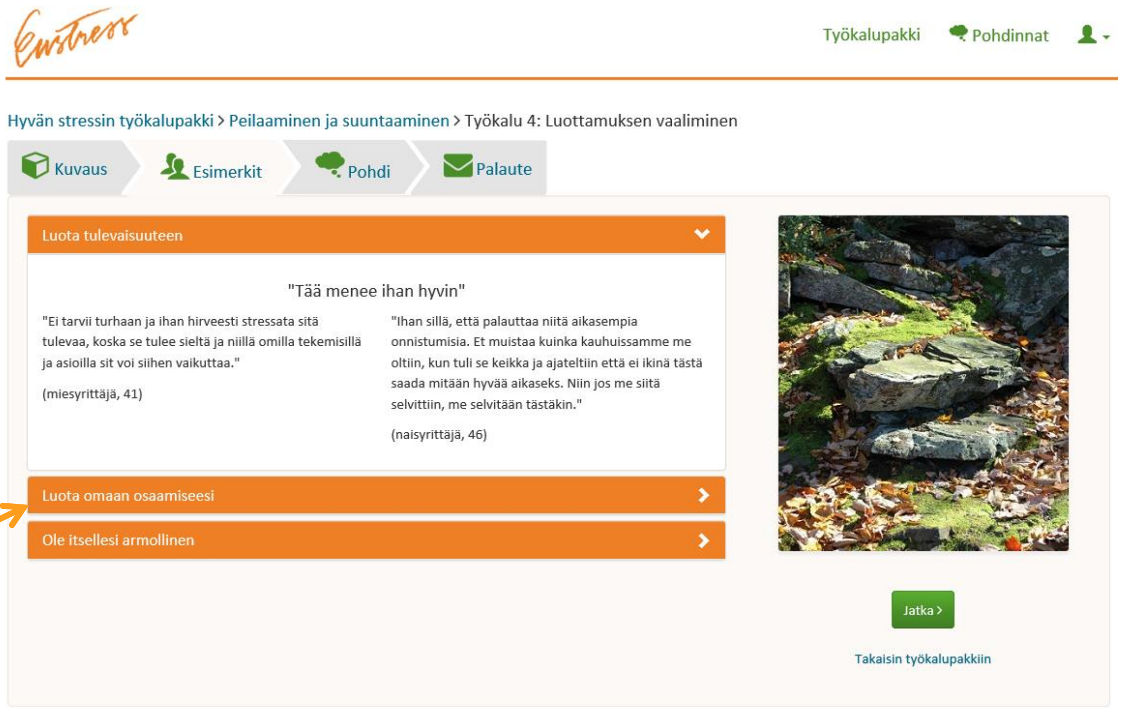

Open more examples by clicking

Content of a tool (3/4): Reflection exercise

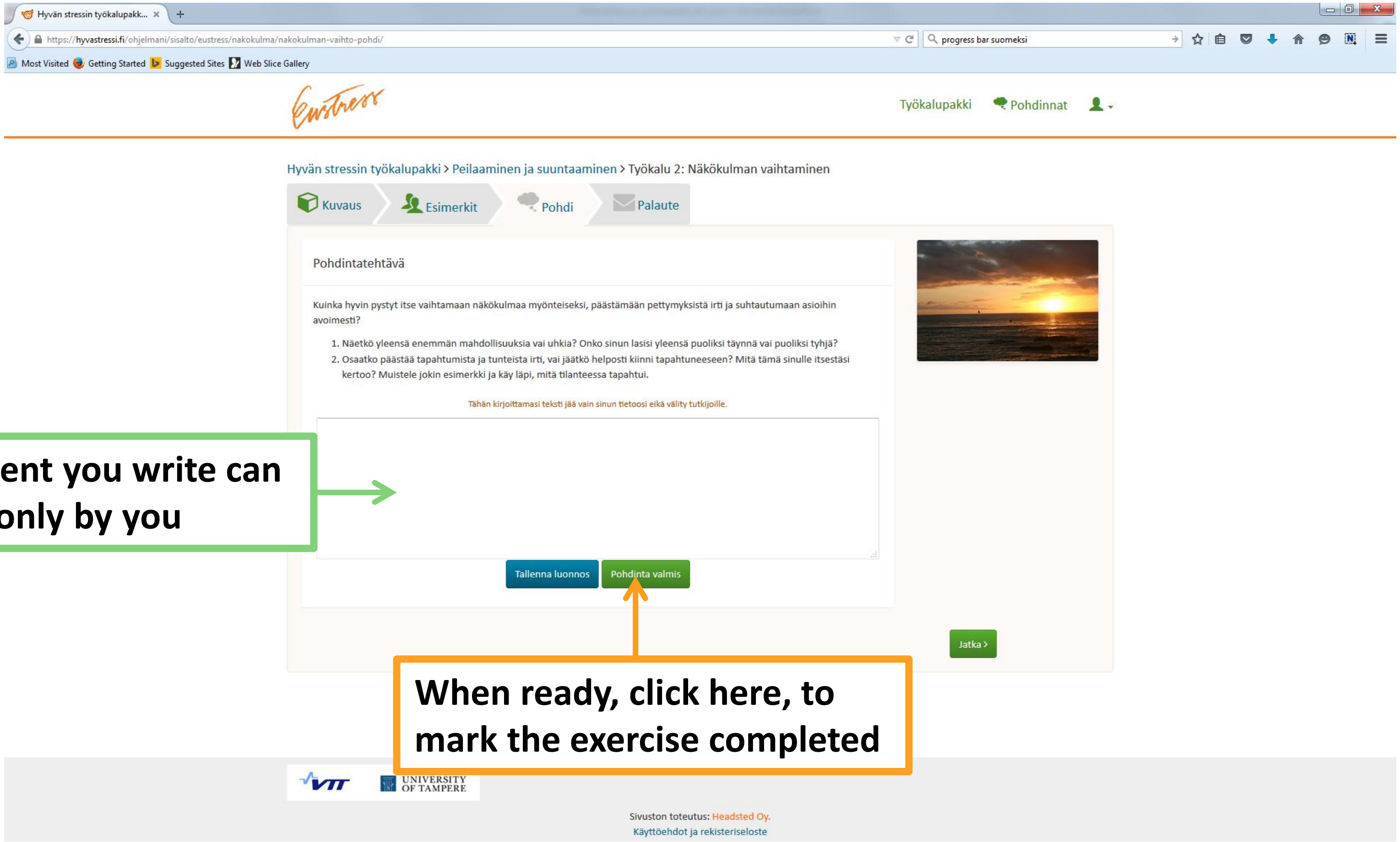

The content you write can be seen only by you

When ready, click here, to mark the exercise completed

Content of a tool (4/4): Feedback to researchers

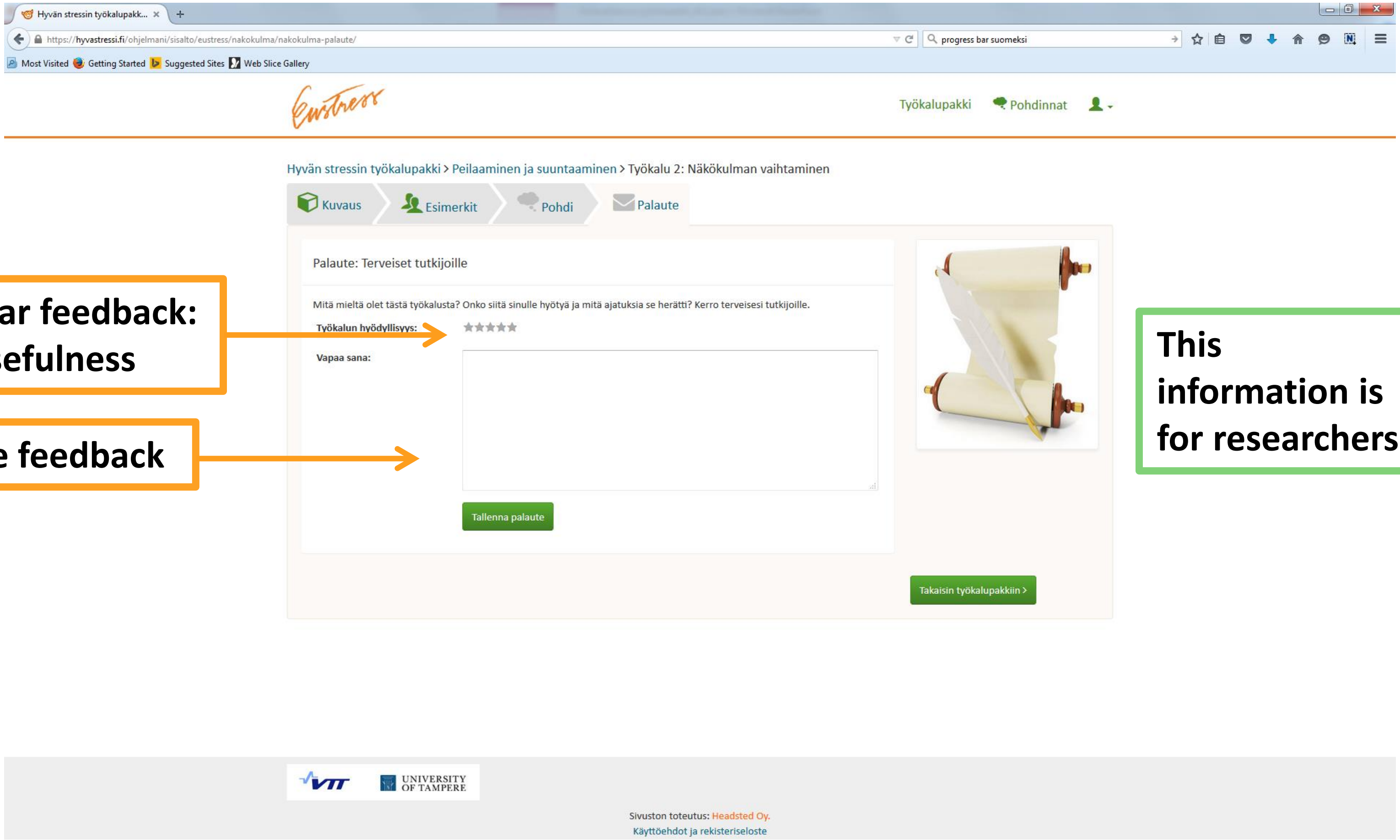

Star feedback: usefulness

Free feedback

This information is for researchers

Eustress

Practise skills in daily life (1/3): Selecting an offline exercise

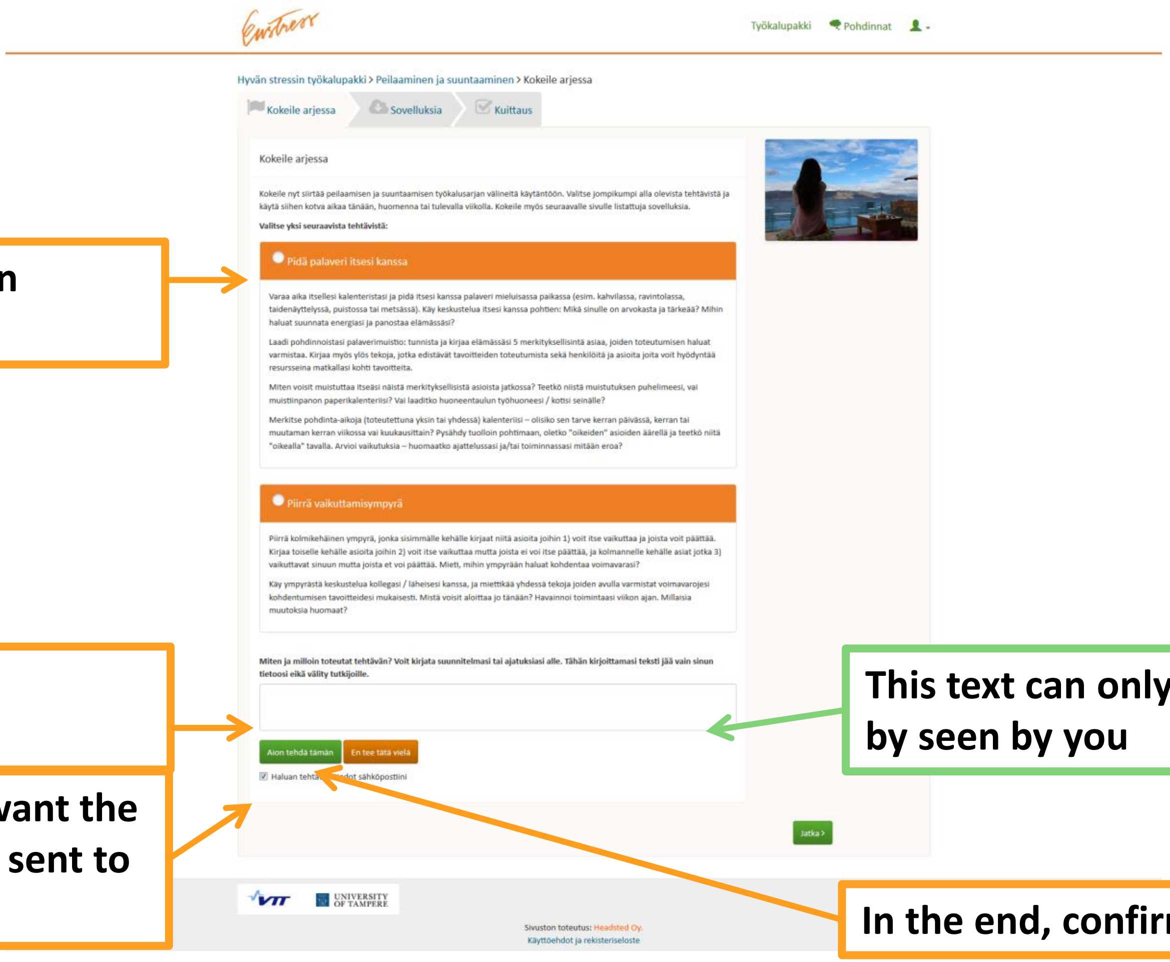

Select an exercise

Your plan and thoughts

Select if you want the exercise to be sent to you by email

This text can only by seen by you

In the end, confirm the selection

Practise skills in daily life (2/3): 3rd party applications

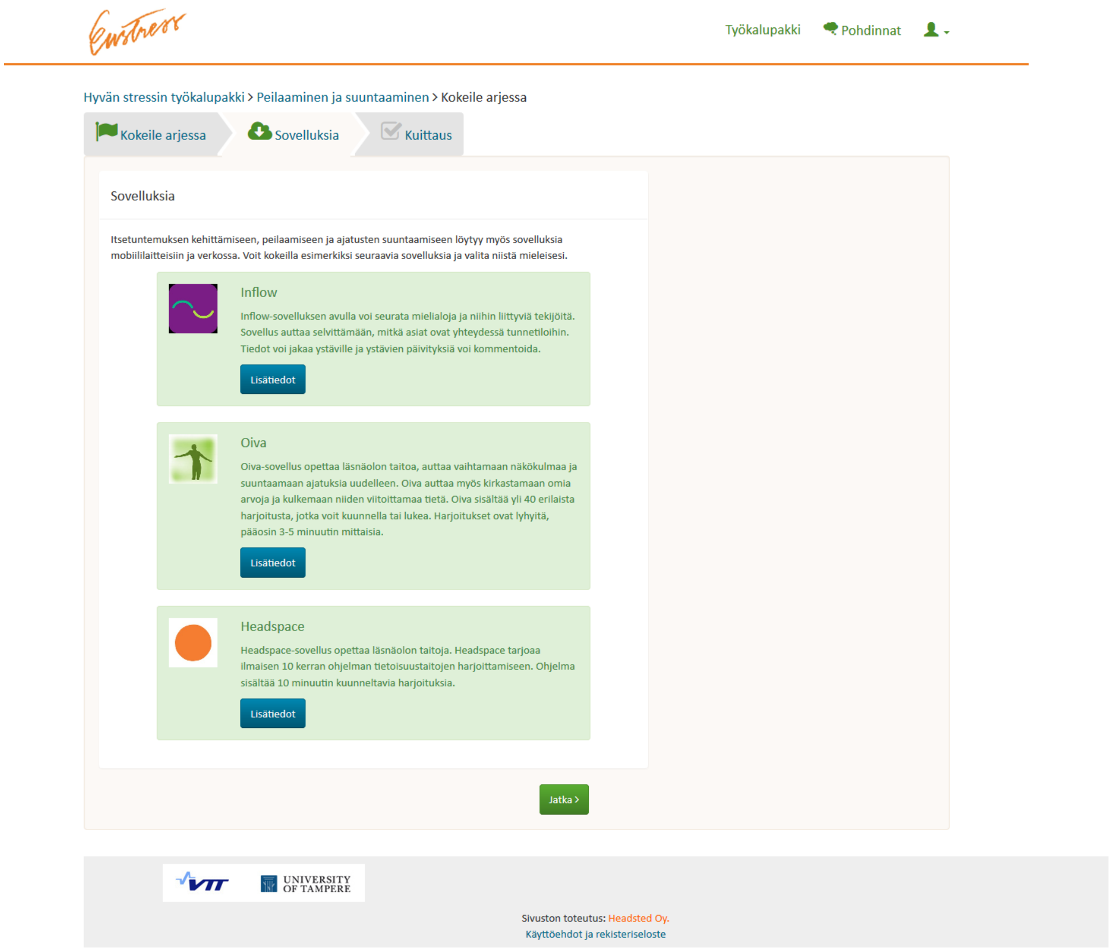

Practise skills in daily life (3/3): Reflection and feedback
(cannot be accessed before 24h from selecting the offline exercise)

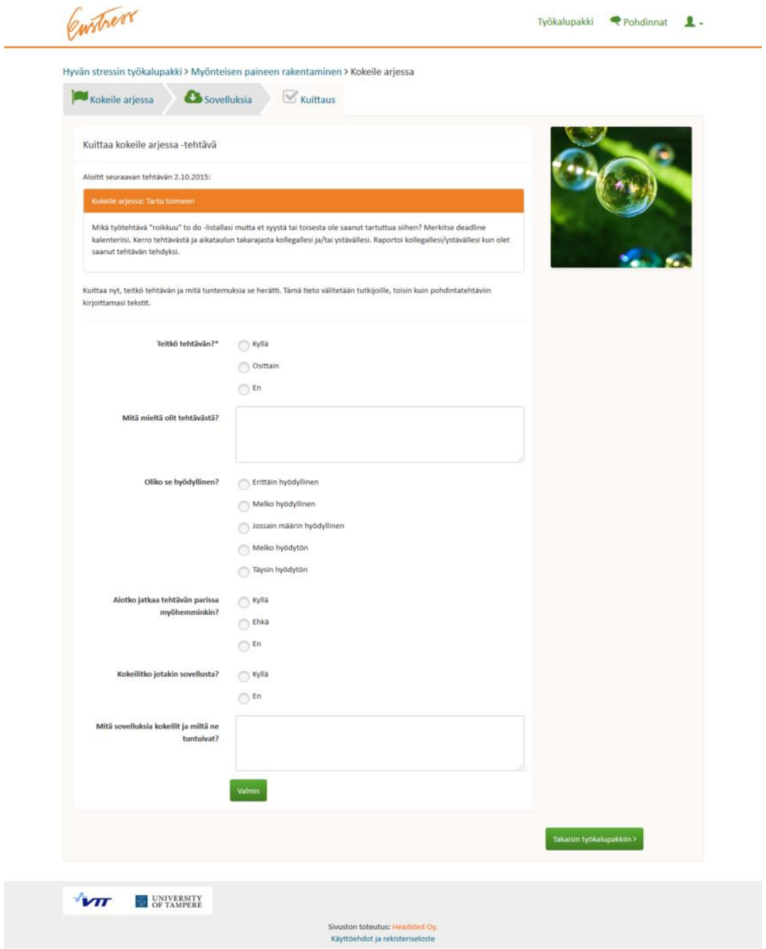

This data is can be seen by researchers

Follow-up questionnaire

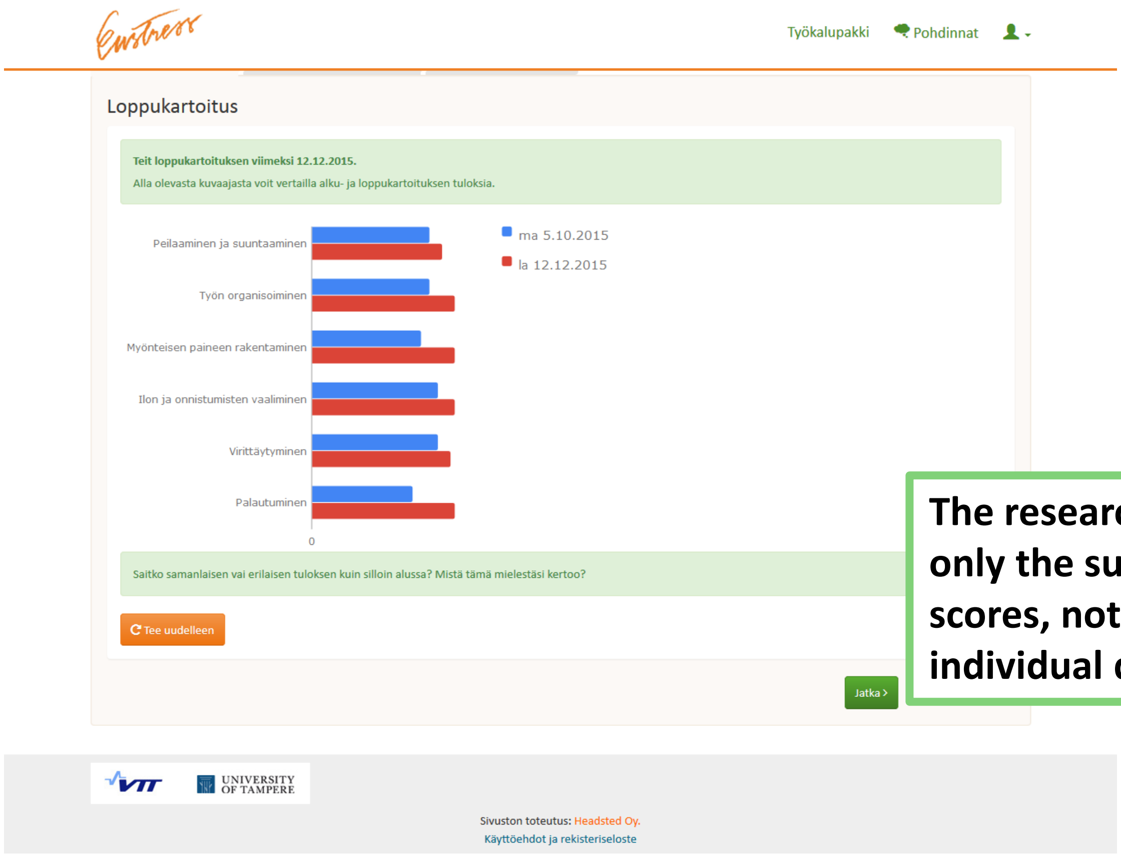

The researchers can see only the summary of the scores, not responses to the individual questions
